# Widespread occurrence of lysine methylation in *Plasmodium falciparum* proteins at asexual blood stages

**DOI:** 10.1038/srep35432

**Published:** 2016-10-20

**Authors:** Inderjeet Kaur, Mohammad Zeeshan, Ekta Saini, Abhinav Kaushik, Asif Mohmmed, Dinesh Gupta, Pawan Malhotra

**Affiliations:** 1 Malaria Biology Group, International Centre for Genetic Engineering and Biotechnology, ICGEB, Aruna Asaf Ali Marg, New Delhi-110067, India; 2 Translational Bioinformatics Group, International Centre for Genetic Engineering and Biotechnology, Aruna Asaf Ali Marg, New Delhi-110067, India; 3 Parasite Cell Biology Group, International Centre for Genetic Engineering and Biotechnology, Aruna Asaf Ali Marg, New Delhi-110067, India

## Abstract

Post-transcriptional and post-translational modifications play a major role in *Plasmodium* life cycle regulation. Lysine methylation of histone proteins is well documented in several organisms, however in recent years lysine methylation of proteins outside histone code is emerging out as an important post-translational modification (PTM). In the present study we have performed global analysis of lysine methylation of proteins in asexual blood stages of *Plasmodium falciparum* development. We immunoprecipitated stage specific *Plasmodium* lysates using anti-methyl lysine specific antibodies that immunostained the asexual blood stage parasites. Using liquid chromatography and tandem mass spectrometry analysis, 570 lysine methylated proteins at three different blood stages were identified. Analysis of the peptide sequences identified 605 methylated sites within 422 proteins. Functional classification of the methylated proteins revealed that the proteins are mainly involved in nucleotide metabolic processes, chromatin organization, transport, homeostatic processes and protein folding. The motif analysis of the methylated lysine peptides reveals novel motifs. Many of the identified lysine methylated proteins are also interacting partners/substrates of PfSET domain proteins as revealed by STRING database analysis. Our findings suggest that the protein methylation at lysine residues is widespread in *Plasmodium* and plays an important regulatory role in diverse set of the parasite pathways.

*Plasmodium falciparum*, a protozoan parasite responsible for the severe form of human malaria, has a complex life cycle in two hosts, namely *Anopheles* mosquito and human. Parasites in these two hosts invade different cell types and propagate in distinct microenvironments. Although transcriptional regulation plays an important role in helping the parasite to adapt to distinct environments, however relatively few regulatory motifs and transcriptional regulators have been reported in *Plasmodium* so far^1^,^2^,^3^. Evidences are emerging to suggest that post-translational modifications (PTMs) play an important role in regulation of fundamental processes of *Plasmodium* growth and host invasion- including cell signaling and epigenetic control of gene regulation. Protein trafficking and interactions between various PTMs are the two very important processes that fine-tune the functions of several *Plasmodium* proteins^4^. Although several PTMs- such as phosphorylation, acetylation, palmotylation, ubiquitylation and lipidation have been identified in *Plasmodium,* however, only phosphorylation/dephosphorylation have been studied extensively^5^,^6^,^7^,^8^,^9^,^10^,^11^.

In the recent years, methylation of proteins has been ranked as the fourth common post-translational modification^12^ and is of common occurrence in human, *Saccharomyces cerevisiae* and Trypanosomes^13^,^14^,^15^,^16^. Protein methylation is mainly found on lysine and arginine residues, although there are reports of methylation of histidine and glutamic acid too^17^. Methylation, particularly lysine methylation is a well-studied phenomenon in histones, which involves addition of one to three methyl groups on the amino acid’s amine group to form mono, di or tri-methyllysine^18^. Histone lysine methylation is involved in transcriptional activation and silencing. The process is regulated by histone lysine methyltransferases (HKMTs) and histone lysine demethylases^19^. Recent proteome-wide lysine methylation studies indicate that the modifications also occur in non-histone proteins such as proteins linked to RNA processing, ribosome assembly, trafficking and signaling^20^,^21^.

Among the apicomplexan parasites, *Plasmodium* and *Toxoplasma* have orthologs of several chromatin remodeling proteins and enzymes responsible for protein methylation and acetylation^22^,^23^. In *P. falciparum,* the histone posttranslational modifications, mainly acetylation and methylation have been shown to play significant role(s) in red blood cell invasion and in virulence gene regulation^24^,^25^. Ten SET domain containing histone lysine methyltransferases (HKMTs), three histone-demethylase orthologs of lysine-specific demethylases (LSD1) and jumonji-C histone demethylases (jHDM) families have been described in *Plasmodium.* These proteins are the targets for novel drug development as the proteins show low sequence similarity to corresponding human counterparts^22^,^26^.

To understand the extent of lysine methylation in blood stage forms of *Plasmodium falciparum,* we analyzed the reactivity of anti-mono/dimethyl lysine and anti-trimethyl lysine antibodies with intact asexual blood stage *Plasmodium* parasites and further immunoprecipitated the *Plasmodium* lysates from the three blood stages, using these antibodies. Intriguingly, the LC-MS/MS analysis of the immunoprecipitates identified several non-histone methylated *Plasmodium* proteins linked with diverse functions such as transport, hemostatic processes and chromosome organization. These results suggest an important role of protein lysine methylation in regulation of various *P. falciparum* biological processes.

## Materials and Methods

### *Plasmodium falciparum* culture

*Plasmodium falciparum* 3D7 was cultured in complete RPMI (1640 (Invitrogen Corporation, USA), 50 mg/L hypoxanthine (Sigma Aldrich Co., USA), 0.5 g/L Albumax I (Gibco, Thermofisher Scientific Inc., USA) and 2 g/L sodium bicarbonate (Sigma Aldrich Co., USA) using O^+^ human erythrocytes (4% haematocrit) under mixed gas (5% O_2_, 5% CO_2_ and 90% N_2_). Cultures were synchronized with 5% sorbitol for at least two successive cycles and harvested using saponin treatment.

### Immunofluorescence assay

Thin smears were prepared from *Plasmodium falciparum* culture at ring, trophozoite and schizont stages. The glass slides were air dried and fixed with pre-chilled absolute methanol for 30 *minutes*. The smears were incubated with 4% BSA in PBS for 2 h at room temperature (RT) to block the non-specific binding. After washing with PBS, the smears were probed with anti-mono/dimethyl lysine polyclonal antibody (abcam; ab23366)(1:20) for overnight at 4 °C, followed by Alexa-Fluor 488 conjugated goat anti-rabbit IgG antibody (A11008; Thermofisher Scientific Inc. USA) (1:200) for 1 h at RT. The slides were washed and mounted with 4′,6-diamidino-2-phenylindoledihydrochloride (DAPI, Molecular Probes, USA) antifade solution (Molecular Probes, USA). Images were captured using a Nikon A1-R confocal microscope.

### Preparation of parasite lysate, Immunoprecipitation and Western blot analysis

Immuno-precipitation was performed using Pierce® Crosslink Immunoprecipitation Kit (Thermofisher Scientific Inc., USA) according to manufacturer’s protocol. Briefly, parasite cell lysates from synchronized cultures of three asexual stages (ring, trophozoite and schizont) were prepared using IP lysis buffer. The stage specific cell lysates were incubated overnight at 4 °C with anti-mono/dimethyl (abcam; ab23366) or anti-trimethyl lysine (Immunechem Pharmaceutical Inc.; ICP0601) antibodies, cross-linked with Protein A/G PlusAgarose by DSS cross-linker. The antibody cross-linked resin was washed with TBS followed by two washes with lysis buffer. Finally, the resin was washed with conditioning buffer and antibody bound proteins were eluted with elution buffer.

Also, the peptides obtained after trypsin digestion of trophozoite stage parasite lysates were lyophilized, desalted and incubated overnight at 4 °C with anti-mono/dimethyl and anti-trimethyl lysine antibodies cross-linked with Protein A/G Plus agarose. The eluted peptides were lyophilized and separated into 12 fractions using hydrophilic liquid interaction chromatography (HILIC) over one hour. Each fraction was separately analyzed on LC-MS/MS.

For western blot analysis, equal amount (50 μg) of total protein from three stages were resolved on 10% SDS-PAGE and transferred onto PVDF membrane (Merck Millipore, Merck KGaA, USA) pre-activated with methanol. The membrane was blocked with 4% BSA and incubated with polyclonal anti-methyllysine antibodies (ImmuneChem Pharmaceuticals Inc, Canada, ICP0601; 1:50) overnight at 4 °C. After three washings with PBST, the membrane was incubated with HRP conjugated anti-rabbit IgG secondary antibody (Sigma Aldrich Pvt. Ltd., USA) for 1 h at RT. The membrane was developed and visualized using SuperSignal West Pico Chemiluminescent Substrate (Thermofisher Scientific Incorporation, USA) as per manufacturer’s instructions.

For confirmation of proteins in the IP experiments, a fraction of IP eluates pulled using anti-methyllysine antibodies were boiled in 4X sample loading buffer, separated on 10% SDS-PAGE gels and transferred to a nitro-cellulose membrane. The membrane was blocked with blocking buffer (ODYSSEY infrared imaging systems; *LI-COR*) and probed with protein specific antisera (anti-PfP12; a 6-cysteine protein and anti-PfTSN; Tudor Staphylococcal Nuclease) using anti-mice IRDye 800CW secondary antibodies (*LI-COR*). Protein bands were imaged in ODYSSEY Infrared Imager (*LI-COR*).

### Tryptic digestion and LC-MS/MS analysis

Eluted proteins from immunoprecipitation were subjected to in-solution digestion. Samples were subjected to subsequent reduction and alkylation of disulfide bonds with 10 mM dithiothreitol (DTT) and 40 mM iodoacetamide for 1 hr. at room temperature. Digestion was performed using trypsin (1:50, enzyme: total protein) (Promega corporation, USA) for overnight at 37 °C and stopped by adding 0.1% trifluoroacetic acid. The samples were cleared by centrifugation at 10000 rpm for 10 min. The digested proteins were concentrated using Speed Vac (Thermofisher Scientific Inc., USA) and analyzed on Orbitrap Velos Pro mass spectrometer coupled with nano-LC 1000 (Thermofisher Scientific Inc., USA). The peptide mixtures were loaded on to a reverse phase C-18 pre-column (Acclaim PepMap, 75 μm × 2 cm, 3 μm, 100 A°, Thermofisher Scientific Inc., USA), in line with an analytical column (Acclaim PepMap, 50 μm × 15 cm, 2 μm, 100 A°). The peptides were separated using a gradient of 5% to 50% of solvent B (0.1% formic acid in 95/5 acetonitrile/water) in 180 min for immunoprecipitates and 120 min for HILIC fractions. The peptides were analyzed in data dependent mode where the precursors were acquired (MS) in Orbitrap at a resolution of 60000 and a minimum of 1000 counts were needed to trigger the MS/MS. Top 20 precursors were allowed to fragment using CID (collision induced dissociation) in Ion trap with collision energy of 35 for the immunoprecipitates. For HILIC fractions, precursors were fragmented using high-energy collision dissociation (HCD) and detected in Orbitrap at a resolution of 7500. Charge state screening of precursors and monoisotopic precursor selection was enabled. Unassigned and singly charged ions were rejected. The parent ions once fragmented were excluded for next 50 s with exclusion mass width of ±10 ppm. The lock mass (m/z 445.120024) enabled the accurate measurement in MS for immunoprecipitated samples and in both MS as well as MS/MS for HILIC fractions. The acquired spectra were analyzed using SEQUEST algorithm in Proteome Discoverer (PD; version 1.4) software, with a precursor tolerance of 20 ppm and tolerance of 0.6 Da for MS/MS for CID and 0.1 Da for HCD against *P. falciparum* database version 10 downloaded from PlasmoDB^27^. Carbamidomethyl (C), Deamidation (NQ) and mono-methyl, di-methyl and trimethyl (K) were set as variable modifications and five missed cleavages were allowed. The resultant identified peptides were validated using Percolator at 5% False Discovery Rate (FDR) (q value < 0.05), which uses PEP (Posterior Error Probability) and q value for validations.

### Lysine methylation motif analysis

The motif search included six amino acid residues N-terminal and C-terminal to each methylation sites. Putative lysine methylation sites were analyzed, based on previously known methyl lysine associated motifs in other organisms, for example LK and MK. To determine the sequence motifs, we developed a PERL script to fetch six amino acids upstream as well as downstream of a central lysine. The frequency of amino acids near lysine residue was also analyzed using WebLogo^28^. To compare neighboring residues in the methylated and non methylated lysines in the proteomics identified proteins, we used Two Sample Logo^29^.

### Gene Ontology (GO) analysis

GO enrichment was performed for functional classification and cellular localization of the methylated proteins using PlasmoDB (version 13.0).

### Interaction analysis using STRING

We downloaded the STRING^30^ Protein-Protein Interaction (PPI) network for *P. falciparum* and converted it into R object using igraph package^31^. Next, we removed the Edges with no experimental evidence of interaction (Experimental < 0). However, the network contained redundant edges and loops which were removed too, using *simplify*() function from igraph package. SET domain containing proteins along with its top 10 nearest neighbors were retrieved and the network was visualized using Cytoscape^32^.

## Results

### Anti-methyl lysine specific antibodies recognize *Plasmodium* proteins at asexual blood stages

**Figure 1:**
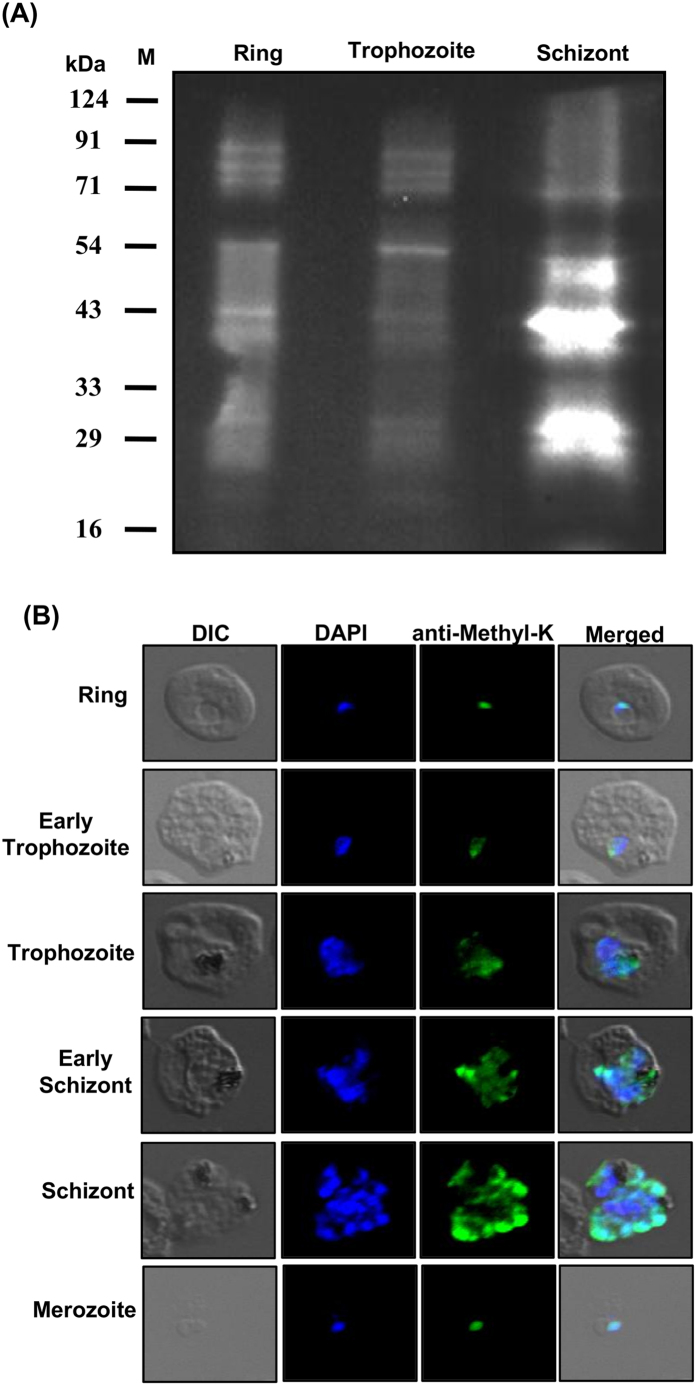
Extensive lysine methylation occurs at asexual blood stages of *P. falciparum*. Anti-methyl-lysine specific antibodies recognize the proteins from three different blood stages of *P. falciparum.* (**A**) Representative western blot showing extent of lysine methylation at ring, trophozoite and schizont stages when the parasite lysates were probed with anti-methyl-lysine specific antibody. (**B**) The same antibodies also immuno-stained the asexual blood stages of the parasite as examined through immuno-fluorescence assay. The representative confocal microscopy images are shown above. DIC- bright field, DAPI-stained nucleus (blue), immunofluorescent cells labeled with anti-Methyl lysine antibody (green), and merged images.

*Plasmodium* genome sequencing analysis has revealed existence of a number of methyltransferases, particularly SET-domain-containing proteins and demethylases that are responsible for lysine methylation in the genome^22^,^33^. To assess the extent of lysine methylation in *Plasmodium* proteins in asexual blood stages, we tested the reactivity of commercially available anti-mono/di methyl lysine and anti-trimethyl lysine antibodies with the parasite lysate and with intact blood stage *P. falciparum* parasites by western blot and immunofluorescence analysis respectively. As shown in Fig. 1A, anti-methyl lysine specific antibodies recognized specific bands at ring, trophozoite and schizont stages, although the extent of reactivity appeared more at the schizont stage. The results were further confirmed by immunofluorescence assay where a considerable staining was observed at all the three parasite blood stages, thereby indicating the extensive methylation of *Plasmodium* proteins at lysine residues. Staining was mainly observed in the periphery of nucleus as blue DAPI stain overlapped with the anti-lysine antibody immunostains, however some staining was also seen in parasite cytoplasm (Fig. 1B).

### Identification of *P. falciparum* lysine methylated proteins

**Figure 2:**
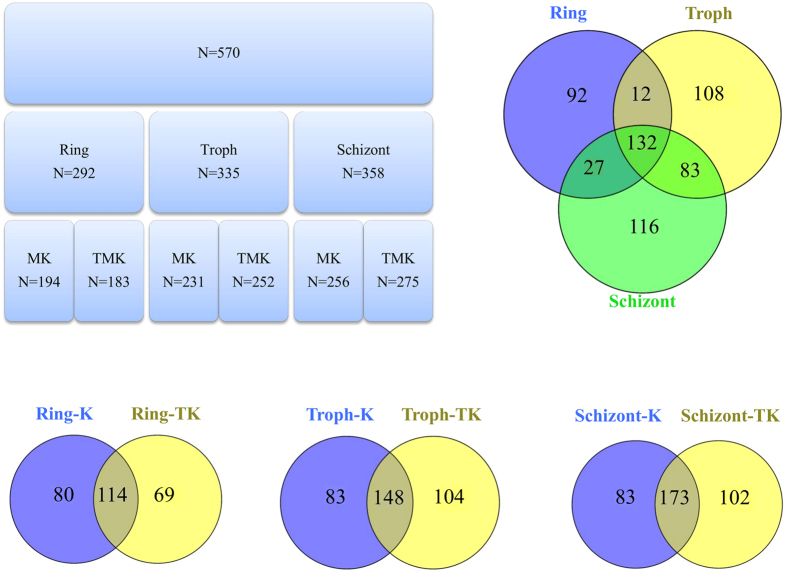
Lysine methylated proteins of *Plasmodiun falciparum*. Anti-methyl-lysine antibodies were used to immunoprecipitate stage specific lysine methylated proteins from *P. falciparum* lysates. N = number of proteins, MK, K = Proteins immunoprecipitated by anti-monomethyl lysine antibody, TMK, TK = Proteins immunoprecipitated by anti-trimethyl lysine antibody.

Next, we carried-out global proteome analysis of lysine methylated *Plasmodium* proteins at asexual blood stages by immunoprecipitating the proteins from *Plasmodium* lysates prepared from the three asexual blood stages; ring, trophozoite and schizont using either anti-mono/di methyl lysine and anti-trimethyl lysine antibodies. Mass spectroscopic analysis of immunoprecipitated proteins identified a total of 570 putative lysine methylated proteins in *P. falciparum* asexual blood stages (Fig. 2). The two antibodies pulled-down approximately the same number of proteins at each stage, with considerable overlap. The one hundred and thirty two proteins identified in the proteome analysis were common to all the three stages (Fig. 2, Supplementary Table 1). To determine whether these putative methylated proteins have methylated lysine residues, we analyzed the spectra of the peptides generated in mass spectrometric analysis. As shown in Table 1, we could identify 364 K-methylated sites on 266 peptides corresponding to *Plasmodium* proteins. The representative spectra of few of the methylated peptides corresponding to *Plasmodium* proteins are shown in Supplementary Fig. 1.

**Figure 3:**
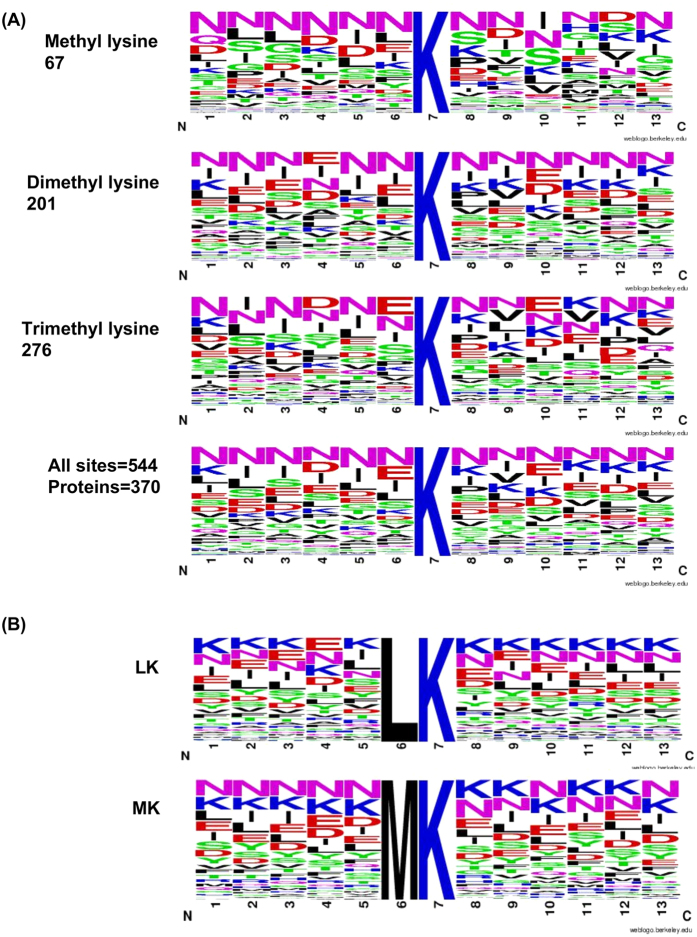
Analysis of identified lysine-methylated proteins for the presence of known motifs. (**A**) Motif representation of methylated lysine sites along with a consensus sequence logo in *P. falciparum*. All the 605 confirmed sites were examined to know the presence of conserved motifs. (**B**) Motif representation of previously reported sites in other organisms.

A number of reports have shown that the pre-fractionation of tryptic peptides either by SCX or HILIC provides a sort of enrichment of methylated peptides that can be better captured on LC-MS/MS for site identification. Between the two methods, HILIC has provided more number of methylation sites as compared to SCX in *S. cerevasiae*^34^. We performed HILIC chromatography analysis on trypsin digested parasite lysate generated from trophozoite stage. Twelve fractions were collected in two replicates and each of the fractions was separately analyzed on LC-MS/MS. As shown in Table 1, we could identify 247 sites in 236 peptides corresponding to *Plasmodium* proteins. Representative spectra of few of these K-methylated peptides are shown in the Supplementary Fig. 2. In total, 605 methylated lysine sites in 502 peptides were identified corresponding to 422 *Plasmodium* proteins. A couple of previous reports in human/mouse cell lines and *Saccharomyces cerevisiae* have shown that many of the lysine methylated sites are associated with EK, LK and MK motifs. We searched the motifs in 570 putative lysine methylated proteins identified by immunoprecipitation of parasite lysates. We could observe LK and EK motifs in many *Plasmodium* proteins (Fig. 3). The “Two Sample Logo” visualization of the residues surrounding methylated and non methylated lysines amongst the proteomics identified proteins reveal that the residues surrounding methylated lysines are different from those surrounding the non methylated lysines (Supplementary Fig. 4). Among the *Plasmodium* methylated peptides, we identified 152 monomethyl, 249 dimethylated and 210 trimethylated sites in the mass spectrometry analysis corresponding to these proteins (Fig. 3).

**Figure 4:**
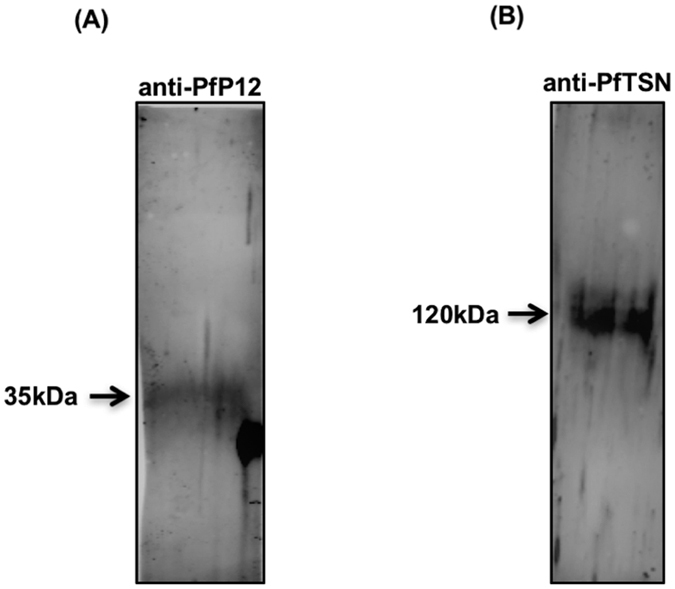
Western blot confirms the presence of methylated lysine proteins in immuno precipitates. Representative western blots (**A**) with anti-PfP12 and (**B**) anti-PfTSN antisera validating the LC-MS/MS results of immunoprecipitation experiments.

To confirm the identity of non-histone lysine methylated proteins, we subjected the immunoprecipitated *Plasmodium* lysate to western blot analysis using anti-PfP12 or anti-PfTSN antisera. As shown in Fig. 4, PfP12 and PfTSN are detected in IP lysate, thus confirming the lysine methylation of the proteins. Altogether, several mass spectrometric analysis runs as well as western blot analysis indicate extensive lysine methylation of *Plasmodium* proteins at asexual blood stages.

### Classification of lysine methylated proteins

**Figure 5:**
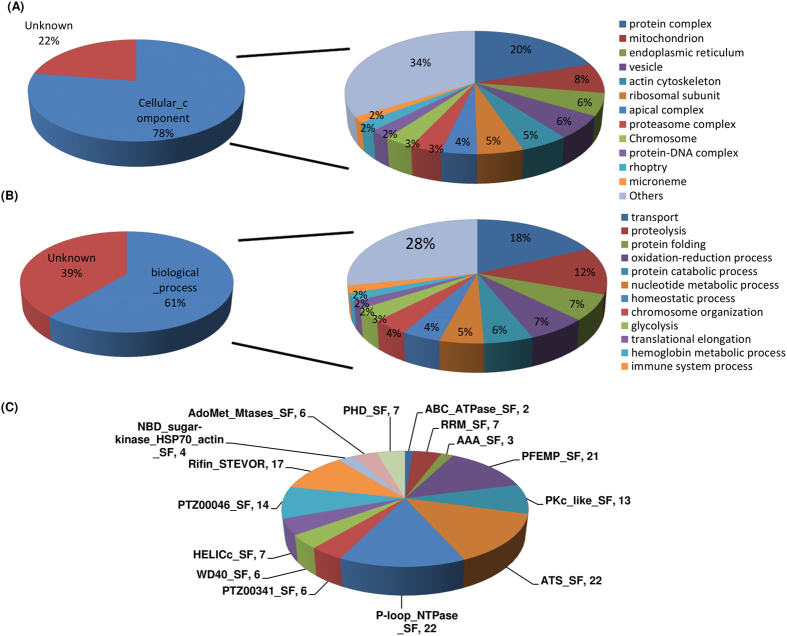
Classification of lysine methylated proteins in *P. falciparum*. (**A**) based on cellular components and (**B**) based on function and (**C**) on the basis of conserved domains. The 570 identified lysine methylated proteins were categorized based on their known or likely functions and cellular localization. Proteins with no annotations in PlasmoDB were categorized as unknown proteins. A pie chart shows the distribution of the proteins based on domain super families.

Mass spectrometry analysis of the anti-methyl lysine antibody immuno-precipitated proteins from the *Plasmodium* lysates suggested that over 10% of the proteins encoded in the *P. falciparum* genome are modified by lysine methylation. To get insight into the role of lysine methylation in the parasite growth and development, we utilized the fully annotated genome database (PlasmoDB) to describe correlations for lysine methylated proteins, their families, subcellular localization and biological function. The identified lysine methylated proteins were classified based on their subcellular localization with GO term analysis using PlasmoDB. As seen in Fig. 5A, we were able to obtain the GO assigned localization of only 78% of the lysine methylated proteins, since a large number of the query proteins were hypothetical. The percentage of proteins with known or predicted subcellular localization was highest for cytoplasmic proteins (Fig. 5A). Many of the identified lysine methylated proteins appeared to be part of protein-protein or protein-DNA complexes associated with chromosome or ribosome.

The diverse localization profile of lysine-methylated proteins suggests that this modification regulates a wide range of cellular functions. To define the functional classes of lysine methylated proteins, we data mined the *P. falciparum* annotated genome database for all identified methylated proteins. Similar to the localization GO terms, we were able to predict functional classes for only 62% of the identified lysine methylated proteins (Fig. 5B). Many of these annotated proteins seem to be associated with the transport/trafficking processes, chromosomal organization and translation regulation (Fig. 5B).

To get insights into the role(s) of lysine methylated *Plasmodium* proteins identified by mass spectrometry analysis, we sorted the proteins based on other conserved domains. As shown in Fig. 5C, several of these proteins belong to major super families such as protein kinase C (PKc), ATS, P-loop_ NTPase, PHD_SF and AdoMet_MTases_SF. The set also included 21 PfEMPs and 17 rifin proteins, indicating a role of lysine methylation in antigenic diversity in *P. falciparum.*

### Lysine methyltransferase-substrate interactome networks and cross talks between PTMs

For further exploration of the breadth of *Plasmodium* protein lysine methylation and associated lysine methyltransferases (KMTs), we generated KMT-substrate networks using STRING database and published literature. These networks are depicted in Supplementary Fig. 3 and are described in Supplementary Table 2. All the *Plasmodium* SET domain proteins show a number of associated partners and many of them are non-histone proteins such as endoplasmin putative (GRP94), bromodomain protein 1 (BP1), proliferating cell nuclear antigen1, guanylyl cyclase (GCalpha). Importantly, some of the lysine-methylated proteins identified in our proteome analysis are also part of this KMTs-substrate network (Supplementary Table 2). For example endoplasmin putative (GRP94) and proliferating cell nuclear antigen 1 (PCNA1) proteins are identified in STRING as well as in our mass spectrometry analysis. We also observed associations among the set domain proteins. For example SET3 protein shows association with SET8 and SET4 proteins in the STRING analysis (Supplementary Table 2).

Extensive cross talk has been reported and predicted between different PTMs in different organisms, including *Plasmodium.* We compared the methylated lysine containing proteins with the previously published studies for phosphorylation and acetylation in *P. falciparum*^5^,^6^,^8^,^35^. This comparison showed that 209 lysine-methylated proteins were phosphorylated too. Further, 173 *Plasmodium* proteins were acetylated as well as lysine methylated and 113 proteins possess all three modifications (Supplementary Table 3). Several proteins from the STRING analysis were either acetylated or phosphorylated (Supplementary Table 2), thus indicating extensive cross talks between various PTMs.

## Discussion

Processes related to intraerythrocytic development of malaria parasite that contributes to malaria associated morbidity and mortality are tightly regulated at transcription as well as translation levels^36^,^37^,^38^. Genome sequencing has shown that basal transcription and translational machineries are conserved in *Plasmodium* parasite, although few recognizable transcription factors have been identified so far^39^,^40^. Like in other eukaryotes, PTMs such as phosphorylation, ubiquitination, sumoylation, acetylation and methylation play an important role in regulating the functions of several *Plasmodium* proteins^4^. In recent times, methylation of proteins has emerged as one of the most prevalent post-translational modifications^41^, in the present study we have examined the lysine methylproteome of *P. falciparum.*

*Plasmodium* genome encodes nine SET domain and two JmjC-domain genes, indicating the presence of protein lysine methylation in malaria parasites^22^. Our current understanding of protein lysine methylation in *Plasmodium* is mainly restricted to histones and its role(s) in regulation of var gene expression. Tri-methylated histone 3 lysine 9 (H3K9me3) has been linked to exclusive expression of certain var genes^42^, while H3K36me3 has been shown to be involved in the repression of these genes^25^. To know the extent of lysine methylation in *Plasmodium* parasites in particularly the non-histone lysine methylation, we performed immunoprecipiataion of *Plasmodium* asexual blood stage lysates with two Methyl lysine specific antibodies and identified lysine methylated proteins by LC-MS/MS analysis. We further validated many of these lysine methylated proteins and also identified additional lysine methylated proteins by HILIC fractionation followed by LC-MS/MS analysis at trophozoite stage. Similar approaches have been previously applied to identify *Saccharomyces cerevisiae* and human lysine methylated proteins^43^,^44^. Based on spectral analysis, we could identify 364 sites in IP analysis and 247 sites in HILIC analysis. In total, we have identified 605 lysine methylated sites in 422 proteins.

A number of previous studies in human and yeast have shown that methylated lysine amino acid residues are part of a motif that has either leucine or methionine at – 1 position^45^,^46^. We could indeed identify leucine residue at -1 position in a number of *Plasmodium* specific methylated peptides identified in our study too. To get functional insights into the roles of methylated lysine containing proteins, especially the non-histone proteins, Gene Ontology (GO) analysis of these *Plasmodium* proteins was performed as described earlier^44^,^46^. Besides histones, we identified a large number proteins involved in transport, protein folding, translational elongation and other important biological processes. For example, lysine-methylated peptides corresponding to a number of ribosome-associated proteins, translation elongation factors, HSPs and DNA mismatch repair proteins were identified in our study. We also identified lysine methylation in several helicases, hydrolases, histone deacetylase complex proteins, kinases and phosphatases. Many of these proteins are methylated in other organisms too^44^,^47^. Remarkably, a number of *Plasmodium* specific proteins are also identified in the present study. These include many surface/secretory proteins; RON3, ROP14, 6-cysteine protein (p12), trophozoite excretory protein (TEX1) Rifin and PfEMP1. Interestingly, two inner membrane complex proteins; 1g and 1c (IMC 1g and 1c) are heavily methylated with 16 and 15 lysine methylation sites, respectively. Intriguingly, many of the lysine methylated proteins, especially the *Plasmodium* surface proteins and proteins involved in gliding motility of merozoites during invasion, have also been shown to be phosphorylated^5^. Summarily, the results suggest an important role of PTMs and their cross talks in the invasion of RBC by merozoites. It is important to mention here that in our analysis we could identify many methylated proteins belonging to PfEMP and Stevor family, whose transcription and antigenic variations have been linked with chromatin/epigenetic memory that includes methylation of histones^25^,^42^,^48^.

To validate our lysine methylome analysis, we used the STRING protein interaction database^49^ to understand the protein interaction networks for PfSET domain proteins and compared the results with the data generated in our methylome analysis. A few common non-histone proteins were identified in the two datasets. In addition, we also examined the proteins identified in STRING analysis for acetylation and phosphorylation. A number of *Plasmodium* proteins showed two or more PTMs, thereby suggesting cross talks among various PTMs. Such cross talks among PTMs such as between methylation and acetylation or between methylation and phosphorylation or between two methylation sites on the same proteins are reported in other organisms too^41^,^44^. Such cross talks probably fine tune the function of individual proteins and elucidation of such cross talks between several PTMs may shed new lights on system biology of this human pathogen. Finally, the data presented here shows that protein lysine methylation is quite wide spread in *P. falciparum* which may be an important gene regulatory processes. However, in order to gain deeper understanding of the role of lysine methylation in *Plasmodium* development and sustenance, a conditional knockdown of each of the nine PfSET domain proteins followed by a quantitative lysine methylome analysis will be required. Additionally, it will be important to experimentally explore each of the nine PfSET domain proteins that may also be important for development of novel chemotherapeutics for malaria. Thus the parasite lysine methylome analysis performed by us is a step forward in elucidating the complex nature of the gene regulatory processes in *Plasmodium,* where only a few transcription factors have been functionally characterized.

## Additional Information

**How to cite this article**: Kaur, I. *et al*. Widespread occurrence of lysine methylation in *Plasmodium falciparum* proteins at asexual blood stages. *Sci. Rep.* **6**, 35432; doi: 10.1038/srep35432 (2016).

## Supplementary Material

Supplementary Information

Supplementary Information

Supplementary Information

## Figures and Tables

**Table 1:** List of the peptides with confirmed methylated lysine residues.

Peptide Sequence	Accession no.	Protein	Modification	Method
KVnGEkGSGEnNDQIITIR	PF3D7_0100100	erythrocyte membrane protein 1, PfEMP1 (VAR)	K6(Dimethyl)	IP
IDIKFGLSDEYGTPIAGPPNIEIPqKISGLVEQADQAAVEVAkDTSQSVAAk	PF3D7_0101600	rifin (RIF)	K43(Dimethyl); K52(Dimethyl)	IP
VLCkITIDYIYMFNPPIK	PF3D7_0103100	vacuolar protein sorting-associated protein 51, putative (VPS51)	K4(Dimethyl)	IP
ITYDLLGYGEMDDVSAYSLSYALNDVETIk	PF3D7_0104200	StAR-related lipid transfer protein	K30(Dimethyl)	HILIC
HNNNDNNNDNDNIcNSSNNNMVAVDINEENNk	PF3D7_0105700	asparagine-rich antigen Pfa35-2	K32(Dimethyl)	HILIC
QTkEDIVTSLPHTSnk	PF3D7_0106500	conserved Plasmodium protein, unknown function	K3(Methyl); K16(Methyl)	IP
KGEDSIIGILAQSSFVFYNWNIRTFIDPYNnFTDDEIVHALkLNGINLGK	PF3D7_0112200	multidrug resistance-associated protein 1 (MRP1)	K42(Dimethyl)	IP
QNEkIEQNEEDK	PF3D7_0113400	Plasmodium exported protein, unknown function	K4(Dimethyl)	IP
kWFEKCITETqNGEIAkK	PF3D7_0115000	surface-associated interspersed protein 1.3 (SURFIN 1.3) (SURF1.3)	K1(Trimethyl); K17(Methyl)	IP
DETADknAANNGEQVMSR	PF3D7_0202000	knob-associated histidine-rich protein (KAHRP)	K6(Trimethyl)	HILIC
EALAIkDkLPGGLDEYQNQLYGICNETCTTcGPAAIDYVPADAPNGYAYGGSAHDGSHGNLr	PF3D7_0202000	knob-associated histidine-rich protein (KAHRP)	K6(Trimethyl); K8(Trimethyl)	IP
LNLcSSkFIHR	PF3D7_0203900	5′-3′ exonuclease, N-terminal resolvase-like domain, putative	K7(Dimethyl)	HILIC
ENNVSnIEEEnIILDSk	PF3D7_0205100	conserved Plasmodium protein, unknown function	K17(Trimethyl)	HILIC
cDAIASDcFLSGnINVEk	PF3D7_0207800	serine repeat antigen 3 (SERA3)	C-Term(Methyl)	IP
AISESGFEHPSEVQqETIPAAITGTDILcQAk	PF3D7_0209800	ATP-dependent RNA helicase UAP56 (UAP56)	K32(Trimethyl)	HILIC
DLVEEEGHAQINnLIINDk	PF3D7_0214800	conserved Plasmodium membrane protein, unknown function	C-Term(Methyl)	IP
NDINNNNNNNNININNNNINNSCSNnYGLk	PF3D7_0218200	conserved Plasmodium protein, unknown function	K30(Dimethyl)	HILIC
EnILEESQVNDDIFNSLVk	PF3D7_0220000	liver stage antigen 3 (LSA3)	K19(Dimethyl)	HILIC
IFAGTYGITnYELIGnk	PF3D7_0301300	alpha/beta hydrolase, putative	C-Term(Methyl)	IP
GVVSFIDSFEHk	PF3D7_0302100	serine/threonine protein kinase (SRPK1)	K12(Dimethyl)	HILIC
KkkkWICNHFHIYSINNDMINYTTTSTHYSILISYNk	PF3D7_0304800	conserved Plasmodium membrane protein, unknown function	K2(Trimethyl); K3(Trimethyl); K4(Dimethyl); K37(Dimethyl)	IP
ELNNTLNk	PF3D7_0305200	conserved Plasmodium protein, unknown function	K8(Dimethyl)	IP
ESNTEDDNIDSEQNSSInMnNSNDDSQNSSNSESNNNDDNDSNNSNEk	PF3D7_0305800	P-loop containing nucleoside triphosphate hydrolase, putative	K48(Trimethyl)	HILIC
HNNNNNNNNNNNNNNNNNNNNNNcCTFk	PF3D7_0309000	dual specificity protein phosphatase (YVH1)	K28(Dimethyl)	HILIC
SCSDCAVIPnLFk	PF3D7_0309500	asparagine synthetase, putative	K13(Dimethyl)	IP
CDSSIDNNGGDNGnGTEGnVnPDTPSCNDNDDDDk	PF3D7_0315200	circumsporozoite- and TRAP-related protein (CTRP)	K35(Dimethyl)	HILIC
NnIHnkPnGYNLK	PF3D7_0317200	cdc2-related protein kinase 4 (CRK4)	K6(Trimethyl)	HILIC
VLDELIVVkALADIPYFDLTEIEGFDFK	PF3D7_0317400	conserved Plasmodium protein, unknown function	K9(Trimethyl)	IP
SSTFSNNSDYSDNSNNSNnSDYSDYSDYSYGInNSYEYVPENSNLk	PF3D7_0317700	CPSF (cleavage and polyadenylation specific factor), subunit A, putative	K46(Methyl)	HILIC
SLCHPGECVGALAAqSIGEPATqMTLNTFHFAGVGSkNVTLGVPRLKELINIVk	PF3D7_0318200	DNA-directed RNA polymerase II subunit RPB1 (RPB1)	K37(Methyl); K54(Trimethyl)	IP
NNMMINNNNNIk	PF3D7_0319400	kinesin-8, putative	K12(Dimethyl)	HILIC
IILTFknIELTNVELKYIINLLMLIVNLk	PF3D7_0319700	ABC transporter I family member 1, putative (ABCI3)	K6(Trimethyl); C-Term(Methyl)	IP
NCNDTnNYDNNSNNNNNNDNHNDNHSNNSGINSSFNNNNNVHNk	PF3D7_0319700	ABC transporter I family member 1, putative (ABCI3)	K44(Methyl)	HILIC
LYSFPNLSTVTSNNFNYHFLIENGIAYIAVFPVTYPkkLAFLFLnDIcK	PF3D7_0320100	protein transport protein SEC22 (SEC22)	K37(Dimethyl); K38(Trimethyl)	IP
TNkPnIVQGQK	PF3D7_0321300	conserved Plasmodium protein, unknown function	K3(Methyl)	HILIC
NNCHGNHNNVcDGNHNNVCDGNHNNICNGNNNNNNNCDSHILTk	PF3D7_0321500	peptidase, putative	K44(Dimethyl)	HILIC
EIcDMEIKKk	PF3D7_0321600	ATP-dependent RNA helicase, putative	C-Term(Methyl)	IP
QIDYFNGPSPkDQGIIMLNNIILkDELkNDLGSLGEKGIVMCDESTNCDYK	PF3D7_0321800	WD repeat-containing protein, putative	K11(Trimethyl); K24(Trimethyl); K28(Methyl)	IP
HDQPGLLSMANAGPnTnSSQFFITLVPcPWLDGk	PF3D7_0322000	peptidyl-prolyl cis-trans isomerase (CYP19A)	K34(Methyl)	HILIC
KPFFFIQLnTDSSQHEk	PF3D7_0402300	reticulocyte binding protein homologue 1 (RH1)	K17(Trimethyl)	HILIC
NSTNQQITLQELKqVqENVEk	PF3D7_0402300	reticulocyte binding protein homologue 1 (RH1)	K21(Dimethyl)	IP
NTQGNSNHDNNnnnNNNNCNNSNSNSTCSSHSk	PF3D7_0403800	alpha/beta hydrolase, putative	K33(Trimethyl)	HILIC
NVDGINNVGDINNAGDTNNAGDINNVGDINnSVDIYnVEHIDEAEkKPnLDNPK	PF3D7_0405300	6-cysteine protein (LISP2)	K46(Dimethyl)	IP
INTLFqk	PF3D7_0405400	pre-mRNA-processing-splicing factor 8, putative (PRPF8)	K7(Dimethyl)	HILIC
InknDGnYYYHNNFSNNSK	PF3D7_0405700	lysine decarboxylase, putative	K3(Dimethyl);	HILIC
IIQKETMNSNANNIYFNNNNNINNINNNNINNNNVYNSHVENIINMKPLIYYGKEk	PF3D7_0407600	conserved Plasmodium protein, unknown function	C-Term(Methyl)	IP
GNINENIFSMnnTYnNMk	PF3D7_0409400	heat shock protein 40 (DnaJ)	K18(Trimethyl)	IP
IHANENGDTNk	PF3D7_0409400	heat shock protein 40 (DnaJ)	K11(Dimethyl)	IP
QVNIYIENSDLnk	PF3D7_0409800	zinc finger protein, putative	C-Term(Methyl)	HILIC
QTPPAPAPAAPPSPPRPLPkPkPPkPDLPPALk	PF3D7_0412900	erythrocyte membrane protein 1, PfEMP1 (VAR)	K20(Trimethyl); K22(Trimethyl); K25(Trimethyl); K33(Dimethyl)	IP
IESESNNQSISSFQnnILSLDIPLDSSLLnGDEEKLk	PF3D7_0413900	ubiquitin carboxyl-terminal hydrolase 13, putative (USP13)	K37(Dimethyl)	IP
DTLESYFSTFGEIDVVQIVLDSSGrSrCFGFVVFSNENSVAkVLk	PF3D7_0414500	RNA-binding protein, putative	K42(Trimethyl); K45(Trimethyl)	IP
NNDNNDNNDNNDNNDnnDnEETLIIQIETk	PF3D7_0415700	conserved Plasmodium protein, unknown function	K30(Dimethyl)	HILIC
MDDDkNDYnPEEEVVTGNWNTPK	PF3D7_0419600	ran binding protein 1, putative	K5(Trimethyl)	HILIC
IqqILPqNGDkPGK	PF3D7_0421300	erythrocyte membrane protein 1, PfEMP1 (VAR)	K11(Methyl)	HILIC
NNNNNNNNNNNNNnNNDNGVk	PF3D7_0423600	conserved Plasmodium protein, unknown function	C-Term(Methyl)	HILIC
QVEEGIkEnDTEGNDK	PF3D7_0500800	mature parasite-infected erythrocyte surface antigen (MESA)	K7(Dimethyl)	HILIC
qVEEGIkENDTESKDK	PF3D7_0500800	mature parasite-infected erythrocyte surface antigen (MESA)	K7(Trimethyl)	HILIC
DIYkPIqSYANNFSK	PF3D7_0501000	Plasmodium exported protein, unknown function	K4(Trimethyl)	HILIC
NGkEEFFGTPDDLISSFFSDMK	PF3D7_0501500	rhoptry-associated protein 3 (RAP3)	K3(Trimethyl)	HILIC
SSSLALVGTnnNDPIFAYCEkDNkSEYYGTPDDLITSFFSIIK	PF3D7_0501600	rhoptry-associated protein 2 (RAP2)	K21(Dimethyl); K24(Dimethyl)	IP
nTSnINNkSICIK	PF3D7_0504700	centrosomal protein CEP120, putative (CEP120)	K8(Dimethyl)	IP
MnIMnKEkTKnK	PF3D7_0504800	conserved Plasmodium protein, unknown function	K8(Trimethyl)	IP
DGLDERGHLIEEEEnDkRDEk	PF3D7_0505100	trafficking protein particle complex subunit 8, putative (TRS85)	K17(Dimethyl); K21(Dimethyl)	IP
DIEqVDDnkEDk	PF3D7_0505700	conserved Plasmodium membrane protein, unknown function	K9(Trimethyl); K12(Trimethyl)	IP
DkDNDIIDEnInNkINYYEKDNIK	PF3D7_0505700	conserved Plasmodium membrane protein, unknown function	K2(Dimethyl); K14(Dimethyl)	IP
QMNkPInkPINkPINKPINKPINKPINKPINKPINK	PF3D7_0508900	conserved Plasmodium protein, unknown function	K4(Dimethyl); K8(Dimethyl); K12(Trimethyl)	HILIC
QMNkPINkPINkPINkPINKPINKPINKPINKPINK	PF3D7_0508900	conserved Plasmodium protein, unknown function	K4(Dimethyl); K8(Trimethyl); K12(Trimethyl); K16(Trimethyl)	IP
MIDLILNNGNkCYQK	PF3D7_0510100	conserved Plasmodium protein, unknown function	K11(Dimethyl)	IP
LPVEISVHFINAGFDTVDTLCTLSnnSLDDVEk	PF3D7_0510300	stripes inner membrane complex protein, putative (SIP)	K33(Dimethyl)	HILIC
MMnAGnDLnSLMYk	PF3D7_0511500	RNA pseudouridylate synthase, putative	C-Term(Methyl)	IP
NNLNGYk	PF3D7_0512500	conserved Plasmodium protein, unknown function	K7(Dimethyl)	IP
ETNNDNINNNDDGNNNnNDDDGNVFITEPLSYNNLKNk	PF3D7_0514300	aspartate–tRNA ligase, putative	C-Term(Methyl)	IP
LFSKDGVLNQGIqIck	PF3D7_0515300	phosphatidylinositol 3-kinase (PI3K)	K16(Trimethyl)	IP
LILYNNFLDIIYEkSNELMNAkNIcLWIYPILSLcK	PF3D7_0516000	RAP protein, putative	K14(Dimethyl); K22(Dimethyl)	IP
ANAIITnLYLDGTLIIEnk	PF3D7_0517500	UTP–glucose-1-phosphate uridylyltransferase, putative	K19(Dimethyl)	HILIC
ANAIITNLYLDGTLIIENk	PF3D7_0517500	UTP–glucose-1-phosphate uridylyltransferase, putative	C-Term(Methyl)	HILIC
VQSINSTkHNNNIK	PF3D7_0518100	RAP protein, putative	K8(Methyl)	HILIC
nETLEEYWWcVESALTWGDGDDNGPDMIVDDGGDATLLVHk	PF3D7_0520900	S-adenosyl-L-homocysteine hydrolase (SAHH)	K41(Methyl)	HILIC
EDkENLNDMYSVNVTNHnDEk	PF3D7_0521300	conserved Plasmodium protein, unknown function	K3(Trimethyl); C-Term(Methyl)	IP
QNLNASNSSGQENkQnESDGK	PF3D7_0524600	50S ribosomal protein L12, apicoplast, putative	K14(Dimethyl)	IP
VPQGALNNIFSICYTSGTTGYPk	PF3D7_0525100	acyl-CoA synthetase (ACS10)	K23(Methyl)	HILIC
DITkYWcAQTNkNNNINNNKIIIPIK	PF3D7_0525500	WD repeat-containing protein, putative	K4(Dimethyl); K12(Dimethyl)	IP
**TVEkIVEVPVYVNR**	**PF3D7_0525800**	**inner membrane complex protein 1g, putative (IMC1g)**	**K4(Trimethyl)**	HILIC
**EVYLEkIVEVPQIK**	**PF3D7_0525800**	inner membrane complex protein 1g, putative (IMC1g)	**K6(Trimethyl)**	IP
**IIYQEkIVEVPQIk**	**PF3D7_0525800**	inner membrane complex protein 1g, putative (IMC1g)	**K6(Trimethyl); K14(Methyl)**	IP
**IIYQEkIVEVPQIK**	**PF3D7_0525800**	inner membrane complex protein 1g, putative (IMC1g)	**K6(Dimethyl)**	IP
**IIYqEkIVEVPQIkTVEK**	**PF3D7_0525800**	inner membrane complex protein 1g, putative (IMC1g)	**K6(Dimethyl); K14(Trimethyl)**	IP
**IIYqEkIVEVPQIKTVEk**	**PF3D7_0525800**	inner membrane complex protein 1g, putative (IMC1g)	**K6(Dimethyl); K18(Trimethyl)**	IP
**IIYQEkIVEVPQIkTVEkIVEVPVYVNR**	**PF3D7_0525800**	inner membrane complex protein 1g, putative (IMC1g)	**K18(Dimethyl)**	IP
**IVEIPKEVYLEkIVEVPQIK**	**PF3D7_0525800**	inner membrane complex protein 1g, putative (IMC1g)	**K12(Trimethyl)**	IP
**IVYVEkVK**	**PF3D7_0525800**	inner membrane complex protein 1g, putative (IMC1g)	**K6(Trimethyl)**	IP
**NVDKIIYQEkIVEVPQIk**	**PF3D7_0525800**	inner membrane complex protein 1g, putative (IMC1g)	**K10(Trimethyl); C-Term(Methyl)**	IP
**NVDKIIYQEkIVEVPqIK**	**PF3D7_0525800**	inner membrane complex protein 1g, putative (IMC1g)	**K10(Dimethyl)**	IP
**NVDKIIYQEkIVEVPQIK**	**PF3D7_0525800**	inner membrane complex protein 1g, putative (IMC1g)	**K10(Trimethyl)**	IP
**NVDkIIYQEKIVEVPQIK**	**PF3D7_0525800**	inner membrane complex protein 1g, putative (IMC1g)	**K4(Dimethyl)**	IP
**TVEkIVEVPVYVNR**	**PF3D7_0525800**	inner membrane complex protein 1g, putative (IMC1g)	**K4(Trimethyl)**	IP
**TVEkIVEVPVYVNR**	**PF3D7_0525800**	inner membrane complex protein 1g, putative (IMC1g)	**K4(Dimethyl)**	IP
DITSIELSNEVLTTnVMDIHNCSLDISk	PF3D7_0526600	conserved Plasmodium protein, unknown function	K28(Methyl)	HILIC
VGLLDYLnk	PF3D7_0529400.2	conserved Plasmodium protein, unknown function	K9(Methyl)	HILIC
NISSFSLDQLVSLCnAYSk	PF3D7_0529700	conserved Plasmodium protein, unknown function	K19(Dimethyl)	HILIC
EIDELNINNSDNIVIIEkLKEELqr	PF3D7_0529800	conserved Plasmodium protein, unknown function	K18(Dimethyl)	IP
DYDkPCPNDYNYLGSVHTDDDEIcAPSSTYEGPCSGEELNIk	PF3D7_0530800	CPW-WPC family protein	K4(Dimethyl); K42(Methyl)	HILIC
VYMGkPFKDVYnEKk	PF3D7_0532300	Plasmodium exported protein (PHISTb), unknown function	K5(Methyl)	IP
CAqnLGGIVAPSSGVLVGIAEGALYAWKPTAITAAk	PF3D7_0600700	rifin (RIF)	K36(Methyl)	IP
LSQEEIEk	PF3D7_0602600	conserved Plasmodium protein, unknown function	K8(Dimethyl)	IP
MkLSqEEIEK	PF3D7_0602600	conserved Plasmodium protein, unknown function	K2(Dimethyl)	IP
EnSTLQnkLSnEIK	PF3D7_0603400	trophozoite exported protein 1 (TEX1)	K8(Trimethyl)	HILIC
ISSIILnSk	PF3D7_0604800	RAP protein, putative	C-Term(Methyl)	HILIC
EYDVYENGLNSCSCIAENSIEISQLInVISKNkIINNNIMLIYIkLIDk	PF3D7_0604800	RAP protein, putative	K33(Methyl); K45(Trimethyl)	IP
QQIREALDLEkGIk	PF3D7_0605500	cyclin dependent kinase binding protein, putative	K11(Dimethyl); K14(Trimethyl)	IP
QQIREALDLEkGIk	PF3D7_0605500	cyclin dependent kinase binding protein, putative	K11(Trimethyl); K14(Dimethyl)	IP
MNInIENILLTNSNkPcINQLIWKLk	PF3D7_0609500	conserved Plasmodium protein, unknown function	K15(Dimethyl); K26(Methyl)	IP
YGYCIIDLSKAcLTnYVLLQk	PF3D7_0609600	hypothetical protein	C-Term(Methyl)	IP
NTNKNNNIQSDTLSVNINnSNDFNk	PF3D7_0611600	conserved Plasmodium protein, unknown function	K25(Trimethyl)	IP
SLNINLNNITkSVADMK	PF3D7_0612200	leucine-rich repeat protein (LRR6)	K11(Dimethyl)	HILIC
TITINGSNGNPSSk	PF3D7_0612700	6-cysteine protein (P12)	K14(Methyl)	HILIC
nPFQnIknINNK	PF3D7_0613300	rhoptry protein ROP14 (ROP14)	K7(Methyl)	HILIC
NnTMDnnNNDENNGHSGDEKSGHHNDDQk	PF3D7_0615600	conserved Plasmodium protein, unknown function	C-Term(Methyl)	IP
TGYFDMLkVENGcMIk	PF3D7_0616900	conserved Plasmodium protein, unknown function	K8(Dimethyl); K16(Methyl)	IP
KVQQIkYASFVLVnLALITLISSIFSINrEYNIPLcVQNDGSSDICMDGK	PF3D7_0618000	conserved Plasmodium membrane protein, unknown function	K6(Trimethyl)	IP
kSSSNSMDSSYNPnMK	PF3D7_0622900	transcription factor with AP2 domain(s), putative (ApiAP2)	K1(Methyl)	IP
NITTNNNnnnNSNNNNSNNNNSNNNNSNNDCVNNEETNFk	PF3D7_0623100	nuclear polyadenylated RNA-binding protein NAB2, putative (NAB2)	K40(Methyl)	HILIC
NLITFDYkQMELFVMAYLSFDEQLLKLLnYSDVFIETAKVLFnTNDVTNELr	PF3D7_0625300	DNA polymerase 1, putative	K8(Dimethyl)	IP
VDLPIISEkDknDILnFAIPMGCNFIAASFIQSADDVR	PF3D7_0626800	pyruvate kinase (PyrK)	K9(Dimethyl); K11(Dimethyl)	IP
DLSSnEEIInISNEKKENDk	PF3D7_0627600	conserved Plasmodium protein, unknown function	K20(Dimethyl)	IP
KNSSNNNnnnTIVDISDGDYTNDEEGTNkPK	PF3D7_0629500	amino acid transporter, putative	K29(Dimethyl)	IP
nNMSNMNNMNNMNNMNNMNNMNNMNNMNNMDSINNVISYSCTNPNMk	PF3D7_0629700	SET domain protein, putative (SET1)	K47(Methyl)	HILIC
NFKcDNCIkCDnCLLHFDSSLK	PF3D7_0629700	SET domain protein, putative (SET1)	K9(Dimethyl)	IP
VnnHTLLTDVCLAAkFEAESLK	PF3D7_0632800	erythrocyte membrane protein 1, PfEMP1 (VAR)	K15(Dimethyl)	HILIC
SINLYkPINPDnGTK	PF3D7_0700100	erythrocyte membrane protein 1, PfEMP1 (VAR)	K6(Methyl)	HILIC
YYDCEINNIKSIIQNEISERIk	PF3D7_0703600	conserved Plasmodium protein, unknown function	K22(Trimethyl)	IP
INqNnILk	PF3D7_0704100	conserved Plasmodium membrane protein, unknown function	K8(Dimethyl)	HILIC
YNITTcDALDNAMDTQNGNIKNDNIKNDNIk	PF3D7_0704200	tRNA m5C-methyltransferase, putative	K31(Dimethyl)	IP
SNLSKLGkLWSIIEPDMIGEIkVFSYnnDLTSNFVETYK	PF3D7_0704400	phosphoinositide-binding protein, putative	K8(Trimethyl); K22(Trimethyl)	IP
GDGECDGEGEGEDDDEDDDDnnnNNk	PF3D7_0704600	E3 ubiquitin-protein ligase (UT)	K26(Trimethyl)	HILIC
LNIITGPnMGGk	PF3D7_0706700	DNA mismatch repair protein MSH2, putative (MSH2-2)	K12(Dimethyl)	HILIC
IIPDkTnnTLTIEDSGIGMTK	PF3D7_0708400	heat shock protein 90 (HSP90)	K5(Trimethyl)	HILIC
IIPDkTNnTLTIEDSGIGMTK	PF3D7_0708400	heat shock protein 90 (HSP90)	K5(Trimethyl);	HILIC
kPEEVTnEEYASFYK	PF3D7_0708400	heat shock protein 90 (HSP90)	K1(Trimethyl)	HILIC
NESDESSnEEGSSTSATSLSFLCEGYDSLDnnk	PF3D7_0708500	heat shock protein 86 family protein	K33(Trimethyl)	HILIC
EDNINNINNVDNNISMVnnVNNVNNVNNDQYk	PF3D7_0709600	ribonucleases P/MRP protein subunit POP1, putative (POP1)	K32(Trimethyl)	IP
nEAEIILSSkQIIGYVSSGGHVLSkGYGYGVAHISFYLFLHNLLNHLFALK	PF3D7_0709600	ribonucleases P/MRP protein subunit POP1, putative (POP1)	K10(Dimethyl); K25(Dimethyl)	IP
LIqVnnYDk	PF3D7_0710000	conserved Plasmodium protein, unknown function	K9(Trimethyl)	HILIC
LNEqINVTLEnk	PF3D7_0710200	conserved Plasmodium protein, unknown function	K12 Dimethyl)	IP
LNEqINVTLEnk	PF3D7_0710200	conserved Plasmodium protein, unknown function	C-Term(Methyl)	IP
SVSLSLSSNEKSSSSFFSTk	PF3D7_0711400	histone deacetylase complex subunit SAP18, putative (SAP18)	K20(Dimethyl)	HILIC
TnITNQk	PF3D7_0713900	conserved Plasmodium protein, unknown function	K7(Dimethyl)	IP
NNnDIINNNISVNk	PF3D7_0715800	drug/metabolite exporter, drug/metabolite transporter	C-Term(Methyl)	IP
NIFENIDVNYILQNINkELLINR	PF3D7_0717600	conserved Plasmodium protein, unknown function	K17(Trimethyl)	HILIC
NIGNVcYLk	PF3D7_0717800	conserved Plasmodium protein, unknown function	K9(Trimethyl)	HILIC
IFIISNLNEILTKIEEnLVLnQNLLIINIYEIYHIDIINNQNMMINANAIINTLk	PF3D7_0718000	dynein heavy chain, putative	K55(Dimethyl)	IP
VESLLNNQPIGGkkr	PF3D7_0720100	small subunit rRNA processing protein, putative	K13(Dimethyl); K14(Dimethyl)	IP
NNNNNNkk	PF3D7_0721000	conserved Plasmodium membrane protein, unknown function	K7(Trimethyl); K8(Dimethyl)	IP
ENSPLNIHNNDDnDDnDDnDENNGDNNNNNDDNNNNNDDNNNk	PF3D7_0721300	DEAD/DEAH box ATP-dependent RNA helicase, putative	K43(Dimethyl)	HILIC
DGLLnFEIDNLNDNkNNDNIGSNk	PF3D7_0723800	conserved Plasmodium protein, unknown function	K15(Methyl); K24(Trimethyl)	IP
NVVTqSnnk	PF3D7_0724000	Rab GTPase activator and protein kinase, putative	K9(Methyl)	IP
ESnnNNTNNVNNNEDINFSNIDETQk	PF3D7_0726300	DNA mismatch repair protein PMS1, putative (PMS1)	K26(Dimethyl)	HILIC
EYIEHIqSVTNNKPSYYk	PF3D7_0726400	conserved Plasmodium membrane protein, unknown function	C-Term(Methyl)	IP
KEDLYIEDENYPSYNIDANSNTLSkVLYkK	PF3D7_0726500	ubiquitin carboxyl-terminal hydrolase, putative	K25(Trimethyl); K29(Trimethyl)	IP
GNLNIDEkVDDVNFncnDFISK	PF3D7_0727800	cation transporting ATPase, putative	K8(Trimethyl)	HILIC
NLLFISFInLFYHk	PF3D7_0728100	conserved Plasmodium membrane protein, unknown function	K14(Dimethyl)	IP
YYIknnIINK	PF3D7_0728700	alpha/beta hydrolase, putative	K4(Methyl)	HILIC
FFqNGDSqPLSGkPVTQSSDK	PF3D7_0800300	erythrocyte membrane protein 1, PfEMP1 (VAR)	K13(Methyl)	HILIC
IPkEHIPMnKPTYQPYINILNEK	PF3D7_0801900	conserved Plasmodium protein, unknown function	K3(Methyl)	HILIC
MLknIFSEYLkSYDnK	PF3D7_0802600	adenylyl cyclase beta (ACbeta)	K3(Dimethyl); K11(Dimethyl)	IP
ENMLCTTLKDqSKkk	PF3D7_0806100	conserved Plasmodium protein, unknown function	K14(Trimethyl); K15(Dimethyl)	IP
YVVAGTqTVVSkSSnAAAqATK	PF3D7_0808800	rifin (RIF)	K12(Trimethyl)	HILIC
EEEGETcTPASPAPAPAPSEDPPVPAPAGDqk	PF3D7_0809100	erythrocyte membrane protein 1, PfEMP1 (VAR)	K32(Methyl)	HILIC
KYNSNNNkNNNNNNNNNDNNDDNNCGNnHGCnnYSEIASSLK	PF3D7_0809200	asparagine-rich antigen Pfa55-14 (pfa55-14)	K8(Trimethyl)	IP
FSILNYDSk	PF3D7_0811900	RNA-binding protein, putative	C-Term(Methyl)	HILIC
TqGTPFDVKVIAILk	PF3D7_0811900	RNA-binding protein, putative	K15(Dimethyl)	IP
YICkIIYRFLEYnFSVTIMDYSCFPINEKMSNKEK	PF3D7_0812100	conserved Plasmodium protein, unknown function	K4(Dimethyl)	HILIC
ESNISNEYDnnINNASINkWSSLKSLNDLNNNDYFENKcISSFNGIIVSILNNMGLIK	PF3D7_0812100	conserved Plasmodium protein, unknown function	K19(Dimethyl)	IP
IYVQVEDGNGcYNIccLQk	PF3D7_0813300	conserved Plasmodium protein, unknown function	K19(Methyl)	HILIC
NMNLNINSGENVLLLGKnGIGk	PF3D7_0813700	ABC transporter F family member 1, putative (ABCF1)	K22(Trimethyl)	IP
GLHQITrCGSTVITDQYVSGQDnSEHVVQEkTVSFIEILLSREQLDMk	PF3D7_0814200	DNA/RNA-binding protein Alba 1 (ALBA1)	K31(Dimethyl); K48(Methyl)	IP
NVDDEEGSSDnSEDnDSSDFDVDIDEENDDTINGNINNGIEk	PF3D7_0816000	ribosome assembly protein RRB1, putative (RRB1)	K42(Dimethyl)	HILIC
NSkEIEnnLk	PF3D7_0817200	conserved Plasmodium protein, unknown function	K3(Dimethyl); K10(Dimethyl)	HILIC
TLVEQcVnnDkDELTVEER	PF3D7_0818200	14-3-3 protein (14-3-3I)	K11(Methyl)	HILIC
TLVEQcVNnDkDELTVEER	PF3D7_0818200	14-3-3 protein (14-3-3I)	K11(Trimethyl)	HILIC
LQPAEIETcMk	PF3D7_0818900	heat shock protein 70 (HSP70)	K11(Methyl)	HILIC
SGVDEkPMIEVTYqGEK	PF3D7_0818900	heat shock protein 70 (HSP70)	K6(Trimethyl);	HILIC
kSqIFTTYADnQPGVLIQVYEGER	PF3D7_0818900	heat shock protein 70 (HSP70)	K1(Dimethyl)	IP
VNGQnVSEQnDISnQk	PF3D7_0820200	phosphatidylglycerophosphate synthase (PGPS)	K16(Trimethyl)	HILIC
DEDGkEEPVLcFVDnQnQK	PF3D7_0821900	conserved Plasmodium protein, unknown function	K5(Dimethyl)	IP
SCYcSEEFAEPcTSEDLVDINNINQIMDNAFCEYNnNDNNNNDNNk	PF3D7_0822700	conserved Plasmodium protein, unknown function	K46(Trimethyl)	HILIC
kNISHSILGCInnLK	PF3D7_0826200	alpha/beta hydrolase, putative	K1(Methyl)	HILIC
IDFVLIGSEIVTDnGGIInk	PF3D7_0828500	translation initiation factor EIF-2b alpha subunit, putative	K20(Dimethyl)	HILIC
HPYkInEEIInIWLnR	PF3D7_0831100	surface-associated interspersed protein 8.1 (SURFIN 8.1) (SURF8.1)	K4(Methyl)	HILIC
ATSGDTHLGGEDFDNk	PF3D7_0831700	heat shock protein 70 (HSP70-x)	K16(Dimethyl)	HILIC
ATSGDTHLGGEDFDNk	PF3D7_0831700	heat shock protein 70 (HSP70-x)	K16(Dimethyl)	IP
IINEPTAAAIAYGLDk	PF3D7_0831700 ;PF3D7_0917900	heat shock protein 70 (HSP70-x)	K16(Dimethyl)	HILIC
DTNVDPEqEEEEEEDDSEEqDATVNGPEDGGGEATqQPQGPSEk	PF3D7_0833500	erythrocyte membrane protein 1, PfEMP1 (VAR)	K44(Trimethyl)	HILIC
CGLAVAkNSYHTcNFGkALkCTELCSQANCSNEATIADGVNKILSTAK	PF3D7_0901100	rifin (RIF)	K7(Trimethyl); K17(Dimethyl); K20(Dimethyl)	IP
LQYQVSCATDPLGETSVLCFYKTIGEPNATAAVVENAkIVVTDAIqEASEVAFk	PF3D7_0901500	rifin (RIF)	K38(Dimethyl); K54(Dimethyl)	IP
nLISPHILNVcTk	PF3D7_0903300	conserved Plasmodium membrane protein, unknown function	C-Term(Methyl)	HILIC
nKINDNNNNNINcDNTk	PF3D7_0903400	DEAD/DEAH box helicase, putative	K17(Dimethyl)	IP
TDLDkSFISDIDInCTnNFNEK	PF3D7_0903800	LCCL domain-containing protein (CCp4)	K5(Dimethyl)	HILIC
KPFFFDMISNMLqkPK	PF3D7_0905100	nucleoporin NUP100/NSP100, putative (NUP100)	K14(Trimethyl)	HILIC
EDNNnEkNEEIEkGK	PF3D7_0906000	exoribonuclease II (RNaseII)	K7(Dimethyl); K13(Dimethyl)	IP
LPNVNNAGILNNPk	PF3D7_0909900	helicase with Zn-finger motif, putative	K14(Trimethyl)	HILIC
LPNVNNAGILNNPk	PF3D7_0909900	helicase with Zn-finger motif, putative	K14(Dimethyl)	HILIC
IIYHSSFDTDMNKkHFVVCSnVcIEnISIITNTVIKLPNVNNAGILNNPK	PF3D7_0909900	helicase with Zn-finger motif, putative	K14(Methyl)	IP
NSNNNNNNNNNLknK	PF3D7_0911100	conserved Plasmodium protein, unknown function	K13(Dimethyl);	HILIC
NkGTNHYIIKNEINNEGESFQELFYK	PF3D7_0912000	conserved Plasmodium protein, unknown function	K2(Methyl)	IP
LSqKMHTLYYNNNIFKNIIqnIPSk	PF3D7_0913900	arginine–tRNA ligase, putative	K25(Dimethyl)	IP
KNNNNNNNnnNIISQNVErLNDENLIKk	PF3D7_0914100	conserved Plasmodium protein, unknown function	C-Term(Methyl)	IP
TLQFLLTNkDDEIEQIK	PF3D7_0914500	conserved Plasmodium protein, unknown function	K9(Trimethyl)	HILIC
DEKNICNNNNNDCNNNNDCNnnnDCNNSCIDk	PF3D7_0916400	conserved Plasmodium protein, unknown function	C-Term(Methyl)	IP
qATkDAGTIAGLNIVR	PF3D7_0917900	heat shock protein 70 (HSP70-2)	K4(Trimethyl)	HILIC
VNALQHFAALPnVELTDVPSSGPMGnk	PF3D7_0918000	glideosome-associated protein 50 (GAP50)	C-Term(Methyl)	HILIC
nAQICINYAFAHFISSGDkTTNLVITAK	PF3D7_0918400	conserved Plasmodium protein, unknown function	K19(Methyl)	HILIC
NPMSSICEQNNEcVLDPNNTCESPGk	PF3D7_0918400	conserved Plasmodium protein, unknown function	K26(Dimethyl)	HILIC
NNLPkPEPLTSVYSFSTSGTYSFG	PF3D7_0919200	PPPDE peptidase, putative	K5(Trimethyl)	HILIC
EFVkASnELIDnDVVFVYVDTISLAKTADNFEIk	PF3D7_0919400	protein disulfide isomerase (PDI9)	K4(Dimethyl); C-Term(Methyl)	IP
SIIEkSLIEnNNNNNNNNNNNNIPK	PF3D7_0919800	TLD domain-containing protein	K5(Trimethyl)	IP
QVSETTLTSQVkNVENFLYLYnFEEIIVLMK	PF3D7_0920400	conserved Plasmodium protein, unknown function	K12(Methyl)	HILIC
kSNSSYSALYTSnNEEEQEDEDEDENEDEDENEDEDENEDEDEEDVENEQNkk	PF3D7_0922100	ubiquitin-like protein, putative	K1(Dimethyl); K52(Trimethyl); K53(Trimethyl)	IP
NNTTTTNNNNNNNNNNNnSGYnnNNSGYNNNNSGHYNIYEEEk	PF3D7_0922100	ubiquitin-like protein, putative	C-Term(Methyl)	IP
LnSYEFSDGk	PF3D7_0922800	conserved Plasmodium protein, unknown function	K10(Dimethyl)	HILIC
SEDSENSKcEEENTDDYMLnFEQIYNSYNNIETTSFFSk	PF3D7_0922800	conserved Plasmodium protein, unknown function	K39(Dimethyl)	IP
kNNINKPQYNEK	PF3D7_0923100	OTU-like cysteine protease, putative	K1(Dimethyl)	IP
nVkEnFGDIYYSFGK	PF3D7_0924200	conserved Plasmodium protein, unknown function	K3(Dimethyl)	HILIC
ENnTILYGnNNNNNNNNNNNNNNNNNNNNNNNNNNNNGcDLLCNIk	PF3D7_0925800	conserved Plasmodium protein, unknown function	K46(Dimethyl)	HILIC
NLENDILk	PF3D7_0926100	protein kinase, putative	K8(Dimethyl)	HILIC
NSCDTAISTMNnqTIDGDTITIILGkK	PF3D7_0929200	RNA-binding protein, putative	K26(Dimethyl)	IP
NSCDTAISTMnnqTIDGDTITIILGKKIIDk	PF3D7_0929200	RNA-binding protein, putative	C-Term(Methyl)	IP
NEHLVSEDPnDDCFInYPLATINLDISDPYkEISEDLIk	PF3D7_0929400	high molecular weight rhoptry protein 2 (RhopH2)	K31(Dimethyl); K39(Dimethyl)	HILIC
NNnnEDEDHLNDLCYSPLLMEDIIk	PF3D7_0934100	TFIIH basal transcription factor complex helicase XPD subunit (XPD)	K25(Dimethyl)	HILIC
IPADQHLTSYSGPSPFEIFGk	PF3D7_0934900	conserved Plasmodium protein, unknown function	K21(Dimethyl)	HILIC
KPAPTAGGEEDQTEk	PF3D7_1000100	erythrocyte membrane protein 1, PfEMP1 (VAR)	C-Term(Methyl)	HILIC
MAAQSSGGGGGCGEEDk	PF3D7_1000100	erythrocyte membrane protein 1, PfEMP1 (VAR)	K17(Methyl)	IP
FnESCMPRPPGSVPGPVIDRAFCDTVDTLVLPSGTGSQTSASTnAVIk	PF3D7_1000200	rifin (RIF)	C-Term(Methyl)	IP
EcLkCAqNLGGIVAPSTGVLGEIAALAVnAWK	PF3D7_1000400	rifin (RIF)	K4(Dimethyl)	IP
GLAAGNAHGMnIVIYHLk	PF3D7_1000500	rifin (RIF)	K18(Trimethyl)	HILIC
EYMTHHVDTKkNNnHnEHHINDNNNNNNIVIIPkDK	PF3D7_1002600	conserved Plasmodium protein, unknown function	K11(Dimethyl); K34(Trimethyl)	IP
**IVEVPQIkEVVR**	**PF3D7_1003600**	inner membrane complex protein 1c, putative (IMC1c)	**K8(Trimethyl)**	HILIC
**TVEVPIIkTVEK**	**PF3D7_1003600**	inner membrane complex protein 1c, putative (IMC1c)	**K8(Trimethyl)**	HILIC
**IEIVEkVVER**	**PF3D7_1003600**	inner membrane complex protein 1c, putative (IMC1c)	**K6(Dimethyl)**	IP
**IEIVEkVVER**	**PF3D7_1003600**	inner membrane complex protein 1c, putative (IMC1c)	**K6(Trimethyl)**	IP
**IIEkWHDKIVEVPQIkEVVR**	**PF3D7_1003600**	inner membrane complex protein 1c, putative (IMC1c)	**K4(Trimethyl); K16(Dimethyl)**	IP
**IIEkWHDkIVEVPQIkEVVr**	**PF3D7_1003600**	inner membrane complex protein 1c, putative (IMC1c)	**K8(Methyl); K16(Methyl)**	IP
**IVEVPQIkEVVR**	**PF3D7_1003600**	inner membrane complex protein 1c, putative (IMC1c)	**K8(Dimethyl)**	IP
**NVTHIVEkIVEVPEVK**	**PF3D7_1003600**	inner membrane complex protein 1c, putative (IMC1c)	**K8(Trimethyl)**	IP
**NVTHIVEkIVEVPEVK**	**PF3D7_1003600**	inner membrane complex protein 1c, putative (IMC1c)	**K8(Dimethyl)**	IP
**TIIQEkIIHVPK**	**PF3D7_1003600**	inner membrane complex protein 1c, putative (IMC1c)	**K6(Trimethyl)**	IP
**TIIQEkIIHVPK**	**PF3D7_1003600**	inner membrane complex protein 1c, putative (IMC1c)	**K6(Dimethyl)**	IP
**TVEVPIIkTVEK**	**PF3D7_1003600**	inner membrane complex protein 1c, putative (IMC1c)	**K8(Trimethyl)**	IP
**YIEkIVEVPHIHYK**	**PF3D7_1003600**	inner membrane complex protein 1c, putative (IMC1c)	**K4(Trimethyl)**	IP
KNYncDDIInIISLIkNHWDIFIGK	PF3D7_1003900	conserved Plasmodium protein, unknown function	K16(Dimethyl)	HILIC
KLnFVTnk	PF3D7_1005100	conserved protein, unknown function	K8(Dimethyl)	IP
QAQMGNFSFPGFNSPVLPTNNNILTnTLDInNkSTLPNIPLYIPNTTSNINNINNINNLLQQPIGNNIIYPPk	PF3D7_1009400	zinc finger protein, putative	K33(Methyl); K73(Trimethyl)	HILIC
HTNIKNHSYLFYIFSFVkNHLQNYPFHHQLIQHINkNANMLQR	PF3D7_1009900	conserved Plasmodium protein, unknown function	K18(Trimethyl); K36(Trimethyl)	IP
MINNNLFVFFLLSFFLSkIcTCLYVTDGSSIILEnIGTkYKLFSTDMK	PF3D7_1010700	dolichyl-phosphate-mannose protein mannosyltransferase, putative	K18(Trimethyl); K39(Trimethyl)	IP
SYcNDQSTGTLEIVSEDLScLk	PF3D7_1012400	hypoxanthine-guanine phosphoribosyltransferase (HGPRT)	C-Term(Methyl)	HILIC
SVSIcIDDSDYIWkEnSScIK	PF3D7_1012700	NLI interacting factor-like phosphatase, putative (NIF4)	K14(Trimethyl)	HILIC
SNILNNELLNTTNNDINKHEEEkDEEHAILNCNIVNNnLLDLSnERIk	PF3D7_1013200	conserved Plasmodium protein, unknown function	K23(Trimethyl); K48(Trimethyl)	IP
NkcTSSSVSSLTNVSSISSNNTMNSDIk	PF3D7_1014200	male gamete fusion factor HAP2, putative (HAP2)	K2(Dimethyl); K28(Trimethyl)	IP
EVEnVKEVEnVKEGENVk	PF3D7_1014300	conserved protein, unknown function	K18(Dimethyl)	IP
DkNCYITSIDYNNNNNNNNNSINNSNNEYTGNNYnnNNk	PF3D7_1015400	conserved Plasmodium protein, unknown function	K2(Trimethyl); C-Term(Methyl)	IP
IAMDVAASEFYnSENkTYDLDFK	PF3D7_1015900	enolase (ENO)	K16(Trimethyl)	HILIC
NADNkEDLTSADPEGQIMR	PF3D7_1016300	glycophorin binding protein (GBP)	K5(Trimethyl)	HILIC
NDEKSEYNYSGNVVEDFnFYMDk	PF3D7_1016600	Plasmodium exported protein (PHISTc), unknown function	K23(Dimethyl)	IP
EDLNNSSSVPTTNINELnk	PF3D7_1018200	serine/threonine protein phosphatase 8, putative (PPP8)	C-Term(Methyl)	IP
NSTWcNNDLMcLPAILTKPYECYEDSSLnLENkVQYPNVYYDSLK	PF3D7_1018300	conserved Plasmodium protein, unknown function	K33(Dimethyl)	HILIC
TKkCIHIIkNNSRqNDK	PF3D7_1018800	conserved protein, unknown function	K3(Dimethyl); K9(Trimethyl)	IP
ILQNIIPLLFSk	PF3D7_1018900	conserved Plasmodium protein, unknown function	K12(Dimethyl)	IP
SLDQEKnKTEIEnTGSKSIPnDSNEGANNk	PF3D7_1021900	conserved Plasmodium protein (10b antigen), unknown function	K30(Dimethyl)	IP
KVTPNSNSNSNSNSSSSSNSSSSNNNHFEk	PF3D7_1022000	RNA-binding protein, putative	K30(Trimethyl)	IP
DVCGLIGYNDISIWKNENCLENIKCIKNTVESYLnTAQEITkGnEEILTk	PF3D7_1023700	conserved Plasmodium protein, unknown function	K42(Dimethyl); K50(Dimethyl)	IP
GDEYDEEDEDEEEEEEDDEnEDDDDDDVEDEDDDEESGECDk	PF3D7_1023900	chromodomain-helicase-DNA-binding protein 1 homolog, putative (CHD1)	K42(Methyl)	HILIC
VHNDFTIPNEYEEk	PF3D7_1024700	conserved Plasmodium protein, unknown function	K14(Dimethyl)	IP
IMnSHFFGLSnETAScCGIMAYMGNRDASk	PF3D7_1025100	glutamine–fructose-6-phosphate aminotransferase [isomerizing], putative (GFPT)	K30(Dimethyl)	IP
MMLQYGNINAQGNYMNGQTNNVMNGQGNNYMNGQTNNVMNGqGNNYMNGqk	PF3D7_1025400	conserved Plasmodium membrane protein, unknown function	K51(Dimethyl)	HILIC
TDkNSASTDADITEK	PF3D7_1027800	60S ribosomal protein L3 (RPL3)	K3(Trimethyl)	HILIC
MDELNkEEIVDNINNEQAK	PF3D7_1028400	nucleolar preribosomal assembly protein, putative	K6(Trimethyl)	HILIC
LIVTSATLDAEk	PF3D7_1030100	pre-mRNA-splicing factor ATP-dependent RNA helicase PRP22, putative (PRP22)	C-Term(Methyl)	HILIC
SNSSDGSSSDGSSSDGSSSDGNSSDGSSSSSSNYk	PF3D7_1032000	ribosome maturation factor RimM, putative (RimM)	K35(Dimethyl)	HILIC
AYCNNGNMDnnTkSNSSDGSSSDGSSSDGSSSDGNSSDGSSSSSSNYk	PF3D7_1032000	ribosome maturation factor RimM, putative (RimM)	K13(Methyl); K48(Trimethyl)	HILIC
DTNNcVIkSYDIDTnDTFFK	PF3D7_1034000	Sec1 family protein, putative	K8(Methyl)	HILIC
FLDLHNAYSRNIKGIIIESLYKLSNTYNTSFENLCKHYLnInIPSINk	PF3D7_1037000	DNA polymerase zeta catalytic subunit, putative	K48(Trimethyl)	IP
IMNSYVEINkSqnMLk	PF3D7_1037000	DNA polymerase zeta catalytic subunit, putative	K10(Methyl)	IP
nIEnIIkLSDGIMIAR	PF3D7_1037100	pyruvate kinase 2 (PyKII)	K7(Dimethyl)	IP
INILNVqnAnDIIk	PF3D7_1038300	conserved Plasmodium protein, unknown function	K14(Trimethyl)	HILIC
MILFNFLInTLLLPHYEnSQnk	PF3D7_1040200	stevor	K22(Dimethyl)	HILIC
nHCDGDGFDCSEIGPNENGSFAIFkCPSCAISCR	PF3D7_1100200	erythrocyte membrane protein 1, PfEMP1 (VAR)	K25(Dimethyl)	IP
MENSDSNkDLQDSK	PF3D7_1103700	casein kinase II beta chain (CK2beta1)	K8(Trimethyl)	HILIC
INPLINDASLVSSFNPPDLk	PF3D7_1103800	CCR4-NOT transcription complex subunit 1, putative (NOT1)	K20(Dimethyl)	HILIC
kNEVNEYLLENNNYEQENNNYGQEKQFVSINTVDIENEILTQk	PF3D7_1104100	syntaxin, Qa-SNARE family (SYN13)	K1(Methyl); K43(Dimethyl)	IP
LSNVFVIGDnTkPYISLPR	PF3D7_1105400	40S ribosomal protein S4, putative	K12(Trimethyl)	HILIC
LPLILNk	PF3D7_1106800	protein kinase, putative	K7(Methyl)	HILIC
nLPLDVLSnnNSSANIk	PF3D7_1106800	protein kinase, putative	K17(Dimethyl)	HILIC
NLPLDVLSNNNSSANIk	PF3D7_1106800	protein kinase, putative	C-Term(Methyl)	HILIC
VnInNDITk	PF3D7_1107300	polyadenylate-binding protein-interacting protein 1, putative (PAIP1)	K9(Dimethyl)	IP
McDEEAEAEEnVEMDGEEDnDDGVNNGk	PF3D7_1107800	transcription factor with AP2 domain(s) (ApiAP2)	K28(Trimethyl)	HILIC
NCTCCk	PF3D7_1107900	mechanosensitive ion channel protein	K6(Dimethyl)	HILIC
IcnkPNLINYLK	PF3D7_1113800	conserved Plasmodium membrane protein, unknown function	K4(Methyl)	HILIC
NPIPSNESQPIISFPNEDDNHAQnEGSInAPSEGEHNNTDNk	PF3D7_1116000	rhoptry neck protein 4 (RON4)	C-Term(Methyl)	IP
NTTQTGnkDTnEMDLENYEDTLNSPK	PF3D7_1116700	dipeptidyl aminopeptidase 1 (DPAP1)	K8(Methyl)	HILIC
SNYnFEkPFLWLAR	PF3D7_1117700	GTP-binding nuclear protein RAN/TC4 (RAN)	K7(Trimethyl)	HILIC
EVFIrELISNSSDAIEk	PF3D7_1118200	heat shock protein 90, putative	K17(Dimethyl)	IP
CLnLkKIYIQLLnEDEK	PF3D7_1118300	insulinase, putative	K5(Dimethyl)	IP
VDNnNNNDDNNNDNNNNNk	PF3D7_1118600	histone acetyltransferase (MYST)	C-Term(Methyl)	IP
QVNNDIETLkK	PF3D7_1120700	conserved Plasmodium protein, unknown function	K10(Dimethyl)	IP
MNNHIcAFrDDTRSkEADEFVr	PF3D7_1121000	palmitoyltransferase, putative (DHHC3)	K15(Trimethyl)	IP
NkNAnEnSNEIETNK	PF3D7_1122900	dynein heavy chain, putative	K2(Dimethyl)	IP
GFNFcISSNKnnLEIVKGNk	PF3D7_1126700	autophagy-related protein 23, putative (ATG23)	C-Term(Methyl)	IP
DVEIkNAVnDVFLLYnAIYK	PF3D7_1128400	geranylgeranyl pyrophosphate synthase, putative (GGPPS)	K5(Dimethyl)	HILIC
MEDNkEENcELNK	PF3D7_1129200	26S proteasome regulatory subunit RPN7, putative (RPN7)	K5(Trimethyl)	HILIC
NkNNNSISINIFGYSLGcSVTLqLVLDIAKSLYNDFFEDIKKVCYEGK	PF3D7_1129300	conserved Plasmodium protein, unknown function	K2(Methyl)	IP
NSqEIIDnk	PF3D7_1129300		K9(Dimethyl)	IP
FNMkPFSYGVDVR	PF3D7_1130200	60S ribosomal protein P0 (PfP0)	K4(Trimethyl)	HILIC
FCNDSLQkLVSnK	PF3D7_1130700	structural maintenance of chromosome protein, putative	K8(Trimethyl)	HILIC
kPLGLIIR	PF3D7_1132700	mitochondrial ribosomal protein L2 precursor	K1(Dimethyl)	HILIC
EEEEDDkNEEVEEQnEEVVEK	PF3D7_1133200	conserved Plasmodium protein, unknown function	K7(Trimethyl);	HILIC
NLTDSknEAETLIYSSEK	PF3D7_1134000	heat shock protein 70 (HSP70-3)	K6(Trimethyl);	HILIC
TQTEINLPFITANqTGPk	PF3D7_1134000	heat shock protein 70 (HSP70-3)	K18(Trimethyl)	HILIC
MSqSkPnqEISSR	PF3D7_1134600	zinc finger protein, putative	K5(Dimethyl)	IP
LPQIYLnHk	PF3D7_1135000.3	conserved Plasmodium protein, unknown function	K9(Dimethyl)	HILIC
NPESkPFCDLIVSGYK	PF3D7_1135800	conserved Plasmodium protein, unknown function	K5(Methyl)	HILIC
TPQSNSDGYIPqCSDDkGr	PF3D7_1136300	tudor staphylococcal nuclease (TSN)	K17(Dimethyl)	IP
SkNNIMDILNnK	PF3D7_1137600	conserved Plasmodium protein, unknown function	K2(Dimethyl)	HILIC
IGLHYGSCVGGVIGSGrLRYDLWGIDVLTGnLMESnGIPGk	PF3D7_1138400	guanylyl cyclase (GCalpha)	K41(Dimethyl)	IP
FEEIPDDPNNSLDNTEnSEHMnTNNNSDQNEk	PF3D7_1138500	protein phosphatase 2C (PPM2)	K32(Methyl)	HILIC
TVFkTInLnNINNK	PF3D7_1138800	WD repeat-containing protein, putative	K4(Dimethyl)	HILIC
TkNTSTqEIDGDTINK	PF3D7_1141100	conserved Plasmodium protein, unknown function	K2(Dimethyl);	IP
KNkPVEYPFAISnK	PF3D7_1142800	conserved Plasmodium protein, unknown function	K3(Dimethyl)	HILIC
DMLQkIEkNYDNNDINNDNNNNDNNNNNDNNNNNNNNNNNNNnNNNQK	PF3D7_1144400	conserved Plasmodium protein, unknown function	K5(Trimethyl); K8(Trimethyl)	IP
NSEIVkILNAPNK	PF3D7_1144800	conserved Plasmodium protein, unknown function	K6(Dimethyl)	IP
IMDEnkETEQTEEGnTEEFVQEK	PF3D7_1149000	antigen 332, DBL-like protein (Pf332)	K6(Methyl)	HILIC
FCLncGFGLGSGVLQSLGLFGGSGIYAWTIGAPAAAIAAAKEAGAAAGIKAGHAVGATkVVELVNSk	PF3D7_1150000	rifin (RIF)	K59(Dimethyl); K67(Trimethyl)	IP
nAESIWQGMLcGLSHAVSArDk	PF3D7_1200400	erythrocyte membrane protein 1, PfEMP1 (VAR)	K22(Trimethyl)	IP
GADDcDDNSNIECk	PF3D7_1200600	erythrocyte membrane protein 1, PfEMP1 (VAR2CSA)	K14(Trimethyl)	HILIC
kFIEDCkGGDGTAGSSWVkR	PF3D7_1200600	erythrocyte membrane protein 1, PfEMP1 (VAR2CSA)	K1(Trimethyl); K7(Trimethyl); K19(Methyl)	IP
MGDDGDDnDDDDDGDDDnNNNNk	PF3D7_1202600	conserved protein, unknown function	K23(Methyl)	IP
nIVnIINCk	PF3D7_1203300	conserved Plasmodium protein, unknown function	K9(Dimethyl)	IP
MIKNIRGTEIqTNNLSMATIENHIDNk	PF3D7_1204900	probable protein, unknown function	K27(Dimethyl)	IP
FKNFGSSYNSYPISYIAFSCVVGIErPELENFVSkLDNAIDYFIkFFk	PF3D7_1205100	O-phosphoseryl-tRNA(Sec) selenium transferase, putative (SEPSECS)	K35(Trimethyl); K45(Trimethyl)	IP
cGNNNYSnSk	PF3D7_1207200	conserved Plasmodium protein, unknown function	K10(Dimethyl)	IP
ISLTEISEPSVLIk	PF3D7_1207700	blood stage antigen 41-3 precursor	K14(Dimethyl)	HILIC
SDDESDnESDDESDnESDDESDDk	PF3D7_1207800	conserved Plasmodium protein, unknown function	K24(Dimethyl)	HILIC
NKNVNVNDNDNGNNNNNSNNNNNNSNGNSATLNNNNNMCVMck	PF3D7_1208200	cysteine repeat modular protein 3 (CRMP3)	K43(Dimethyl)	HILIC
TIALQNICGLnLk	PF3D7_1208700	conserved protein, unknown function	K13(Dimethyl)	HILIC
NVGqNDDTLNNNNNNNINSVNNNNNHVVGGLHqTQTCEGk	PF3D7_1209400	cytosolic iron-sulfur protein assembly protein 1, putative (CIA1)	K40(Trimethyl)	HILIC
IIEqAkLKHNqIYNKELHNLAr	PF3D7_1211300	DNA helicase MCM8, putative (MCM8)	K6(Dimethyl)	IP
YYDHNNMCGDNNICDDNNIcGDNEIYGDNk	PF3D7_1218200	conserved Plasmodium protein, unknown function	K30(Trimethyl)	HILIC
NNSkEnINFIK	PF3D7_1218700	conserved Plasmodium protein, unknown function	K4(Dimethyl)	HILIC
NNTIINMYNQNIrHSNSNnnTINDMNNNNINk	PF3D7_1220000	conserved Plasmodium protein, unknown function	K32(Dimethyl)	IP
WDHVDInEDEnNk	PF3D7_1220300	cell cycle associated protein, putative	K13(Dimethyl)	HILIC
IDGNILDNNKIDGnILDnnkIDGNILDNNK	PF3D7_1221000	histone-lysine N-methyltransferase, H3 lysine-4 specific (SET10)	K20(Trimethyl)	IP
DMVnDPnYDSVkVEETDDPNK	PF3D7_1222300	endoplasmin, putative (GRP94)	K12(Methyl)	HILIC
DMVnDPNYDSVkVEETDDPNKK	PF3D7_1222300	endoplasmin, putative (GRP94)	K12(Trimethyl)	HILIC
ILLTDnYnk	PF3D7_1223300	DNA gyrase subunit A (GyrA)	K9(Trimethyl)	HILIC
FNDLqkGnEQEK	PF3D7_1223300	DNA gyrase subunit A (GyrA)	K6(Trimethyl)	IP
nTEDEnnTSSSYLFSLSFQNSGk	PF3D7_1224400	conserved Plasmodium protein, unknown function	K23(Dimethyl)	HILIC
ENVNKInnNNNNNNkk	PF3D7_1225900	conserved Plasmodium protein, unknown function	K15(Dimethyl); K16(Trimethyl)	IP
NIVnQLFNYISk	PF3D7_1227200	potassium channel (K1)	K12(Trimethyl)	HILIC
KNVIDNNIYk	PF3D7_1227300	conserved Plasmodium protein, unknown function	K10(Methyl)	HILIC
NKYYkDNIYDGNnIcDGnNIYCNNNNICCNNNNICCNNNNIYCNNNNIYDNNTCDK	PF3D7_1227400	conserved Plasmodium protein, unknown function	K5(Methyl)	HILIC
NKYYkDNIYDGNNICDGNnIYCNNNNICCNNNNICCNNNNIYCNNNNIYDNNTCDk	PF3D7_1227400	conserved Plasmodium protein, unknown function	K5(Dimethyl); K56(Dimethyl)	HILIC
ELnTSYDnnSPTDSTYk	PF3D7_1231600	pre-mRNA-splicing factor ATP-dependent RNA helicase PRP2, putative (PRP2)	C-Term(Methyl)	IP
TVYLFDIFLNEqSk	PF3D7_1232000	phenylalanine–tRNA ligase (aFRS)	C-Term(Methyl)	HILIC
GMESDnINEMVSDNINEMASDNINEMVSDNINEMTSDNINkMANQMNYEQNTDGIIIk	PF3D7_1233600	asparagine and aspartate rich protein 1 (AARP1)	K41(Dimethyl); C-Term(Methyl)	IP
LNVqRDKTFnEEDnIk	PF3D7_1234100	bromodomain protein, putative	K16(Dimethyl)	IP
NNNEPFSTLNLECNTk	PF3D7_1235200	V-type K+-independent H+-translocating inorganic pyrophosphatase (VP2)	K16(Dimethyl)	HILIC
GDDDDDDDDDDDDDDDDDDDnDDDDDDDDDDDDDDDGDNQITk	PF3D7_1237200	conserved Plasmodium protein, unknown function	K43(Trimethyl)	HILIC
VNVDNICIPNk	PF3D7_1237500	conserved Plasmodium protein, unknown function	K11(Dimethyl)	HILIC
TGDIITnDDSLTNNLCIFk	PF3D7_1237500	conserved Plasmodium protein, unknown function	K19(Methyl)	IP
FLEnEDkVDLEIDkVDELLYFEEIK	PF3D7_1238800	acyl-CoA synthetase (ACS11)	K7(Dimethyl); K14(Dimethyl)	IP
AIGDAnSErHPcGIGk	PF3D7_1240400	erythrocyte membrane protein 1, PfEMP1 (VAR)	K16(Trimethyl)	IP
NDNNGDDYkEDNYDDDDDDDDDDDEk	PF3D7_1245600	kinesin, putative	K9(Methyl); K26(Methyl)	IP
VnInGNVNNk	PF3D7_1246900	RAC-beta serine/threonine protein kinase (PKB)	C-Term(Methyl)	HILIC
IINQEkPR	PF3D7_1247800	dipeptidyl aminopeptidase 2 (DPAP2)	K6(Dimethyl)	HILIC
CNNDNITEEGENMNVQnLESLQnDGNGVIELGCGLGQISk	PF3D7_1249900	apicoplast dimethyladenosine synthase, putative	K40(Methyl)	HILIC
AHLLqqNqLQGLTHkINFENNLGK	PF3D7_1250100	osmiophilic body protein (G377)	K15(Trimethyl)	IP
VAMATAEkVGIQLGIDAGNAAGIK	PF3D7_1254200	rifin (RIF)	K8(Methyl)	HILIC
DFYqQLQSGYGDVNAFLELLnkETTcK	PF3D7_1300100	erythrocyte membrane protein 1, PfEMP1 (VAR)	K22(Trimethyl)	IP
GILDINDPSVTNnVnEVHDASNTQGSVSNTSDITNGHSESSLNRTTNAQDIk	PF3D7_1301600	erythrocyte binding antigen-140 (EBA140)	C-Term(Methyl)	IP
DAVFSLPPTnEk	PF3D7_1302800	40S ribosomal protein S7, putative	K12(Dimethyl)	IP
NENIVDMVkPYDDFCkEIEYNYFIPIQILYK	PF3D7_1306500	MORN repeat protein, putative	K9(Trimethyl); K16(Dimethyl)	HILIC
AVGYNNNYLNNNNNMnSAVnNNSSNGNNMk	PF3D7_1312900	eukaryotic translation initation factor 4 gamma (EIF4G)	C-Term(Methyl)	HILIC
NPLYYVSLLFNkPNSFPYLIK	PF3D7_1313100	conserved Plasmodium protein, unknown function	K12(Methyl)	HILIC
LSLFYnknSInNIK	PF3D7_1317200	transcription factor with AP2 domain(s) (ApiAP2)	K7(Trimethyl);	HILIC
NELGnnECNNNNNNYISk	PF3D7_1317200	transcription factor with AP2 domain(s) (ApiAP2)	K18(Dimethyl)	HILIC
NVLkNIYNNNIINNNDnILnk	PF3D7_1319600	conserved Plasmodium protein, unknown function	K4(Dimethyl); K21(Trimethyl)	IP
QNSYDISEVSINCYYDDVIkCYMDYTMHGMEDETnFYLCEFCEQnIFDMNNMIK	PF3D7_1322100	variant-silencing SET protein (SETvs)	K20(Dimethyl)	HILIC
QINDTINk	PF3D7_1322100	variant-silencing SET protein (SETvs)	K8(Dimethyl)	IP
GcDNADNDDDNNDDNNDGDNNNDDDNNNDDNNIDDNDGDnnNDEk	PF3D7_1324000	conserved Plasmodium protein, unknown function	K45(Methyl)	HILIC
nDDTIVkNYMNNIENIk	PF3D7_1324500	DEAD box helicase, putative	K7(Dimethyl); C-Term(Methyl)	IP
ALDTSHTNVMAYSNck	PF3D7_1324900	L-lactate dehydrogenase (LDH)	K16(Methyl)	HILIC
QMFNVHPYDSFDNDIGkGnTnIICk	PF3D7_1326600	conserved Plasmodium protein, unknown function	K17(Dimethyl); K25(Dimethyl)	IP
KPSIFIkPLSnSPK	PF3D7_1327300	conserved Plasmodium protein, unknown function	K7(Trimethyl)	HILIC
GILFLFILIGFVLkkPDEIkPLLk	PF3D7_1328900	conserved Plasmodium protein, unknown function	K14(Dimethyl); K15(Dimethyl); K20(Trimethyl); K24(Methyl)	IP
NIFEnNDLSPLrk	PF3D7_1334100	conserved Plasmodium protein, unknown function	K13(Dimethyl)	IP
LYNLGDVFNHVVDISnkk	PF3D7_1335100	merozoite surface protein 7 (MSP7)	K17(Dimethyl); K18(Dimethyl)	IP
NEQEISTQGQEVQkPAqGGESTFQk	PF3D7_1335100	merozoite surface protein 7 (MSP7)	K14(Methyl); K25(Trimethyl)	IP
NEQEISTQGQEVQkPAqGGESTFqk	PF3D7_1335100	merozoite surface protein 7 (MSP7)	K14(Dimethyl); K25(Dimethyl)	IP
EIYVLYnKLLnLnk	PF3D7_1335800	conserved Plasmodium protein, unknown function	C-Term(Methyl)	IP
NILLNGGNDLSk	PF3D7_1336000	conserved Plasmodium protein, unknown function	K12(Dimethyl)	IP
LPVLVTSHGSIFESNAVck	PF3D7_1338300	elongation factor 1-gamma, putative	K19(Trimethyl)	HILIC
HINNNIIDIDInInEk	PF3D7_1338500	conserved Plasmodium protein, unknown function	K16(Dimethyl)	HILIC
EkNNNIVHInINK	PF3D7_1339700	conserved Plasmodium protein, unknown function	K2(Trimethyl)	HILIC
EkNNNIVHInINK	PF3D7_1339700	conserved Plasmodium protein, unknown function	K2(Trimethyl)	HILIC
SANInITnHnINVQMNDSMNGHLVDEk	PF3D7_1342900	transcription factor with AP2 domain(s) (ApiAP2)	K27(Dimethyl)	HILIC
EnKLIDLWk	PF3D7_1343300	conserved Plasmodium protein, unknown function	K9(Trimethyl)	IP
KENFLDAAnLInDDSGLNNLk	PF3D7_1343700	kelch protein K13 (K13)	K21(Dimethyl)	IP
NIPQLNENGNSNSNGNSnGNSNGNSNGNSNGNSNGNSNk	PF3D7_1343800	conserved Plasmodium protein, unknown function	K39(Methyl)	HILIC
LTKNIkHEYIINPNYFEGYVEIYLSLIPTDHGERPFSRcFSSWGIqr	PF3D7_1343800	conserved Plasmodium protein, unknown function	K6(Methyl)	IP
ILCnnnNk	PF3D7_1343900	U4/U6 small nuclear ribonucleoprotein PRP4, putative (PRPF4)	K8(Dimethyl)	IP
KnVDIAVSSSSkPIINAGNGTGEHPTQSLLDFYTIHNYFPFILDRNINK	PF3D7_1344800	aspartate carbamoyltransferase (ATCase)	K12(Trimethyl)	IP
NEEqKEnPDnk	PF3D7_1345800	conserved Plasmodium protein, unknown function	K11(Trimethyl)	IP
AVNEnGEkPDEEVK	PF3D7_1347500	DNA/RNA-binding protein Alba 4 (ALBA4)	K8(Trimethyl)	HILIC
NDDDIYDCNNESTIDHSNNNNNNNNVYYNNTNIYNNQDLSk	PF3D7_1348400	conserved Plasmodium membrane protein, unknown function	K41(Trimethyl)	HILIC
GNVNCNIEHIIMNEHMMEYIDFScLnYDEk	PF3D7_1349500	conserved Plasmodium protein, unknown function	C-Term(Methyl)	HILIC
InNNNNNNk	PF3D7_1350400	ubiquitin-activating enzyme E1, putative	K9(Dimethyl)	IP
nYITSEnFNDk	PF3D7_1351000	phosphatidylinositol transfer protein, putative	K11(Trimethyl)	IP
SYnLLYQNEAk	PF3D7_1352300	conserved Plasmodium protein, unknown function	K11(Trimethyl)	IP
nkDnISDINIIEK	PF3D7_1353400	Ran-binding protein, putative	K2(Methyl)	HILIC
EISSEIYLVGLNFLGkKVDK	PF3D7_1354300	large subunit rRNA methyltransferase, putative	K16(Trimethyl)	IP
RAMDFknEELMkIQK	PF3D7_1354300	large subunit rRNA methyltransferase, putative	K6(Trimethyl); K12(Trimethyl)	IP
MEnVLVDHkDnAk	PF3D7_1356800	serine/threonine protein kinase, putative (ARK3)	K9(Trimethyl); K13(Dimethyl)	IP
SQAAGqAYLEAk	PF3D7_1356800	serine/threonine protein kinase, putative (ARK3)	K12(Dimethyl)	IP
NVSVkEIK	PF3D7_1357100	elongation factor 1-alpha	K5(Trimethyl)	IP
NVSVkEIK	PF3D7_1357100	elongation factor 1-alpha	K5(Trimethyl)	IP
TYHTLNNLSLNSnnNNk	PF3D7_1359300	exosome complex exonuclease RRP44 (DIS3)	K17(Trimethyl)	IP
ILEALLVCISILLLTFGVYYEkNknMIDICTHFCSNPYLSINNLDHMNISCLCk	PF3D7_1360500	guanylyl cyclase beta (GCbeta)	K22(Dimethyl); K24(Dimethyl); C-Term(Methyl)	IP
FTTkGIVGDAEVALkPr	PF3D7_1361900	proliferating cell nuclear antigen 1 (PCNA1)	K4(Trimethyl); K15(Dimethyl)	IP
ALGEDTPFTHISGSEVYSLEMSk	PF3D7_1362200	RuvB-like helicase 3 (RUVB3)	K23(Trimethyl)	HILIC
qEkNTKKAEIVYSI	PF3D7_1363300	50S ribosomal protein L9, mitochondrial, putative	K3(Methyl)	HILIC
FDIPLLGDNTATSIIGLNIKNEDk	PF3D7_1364000	conserved Plasmodium protein, unknown function	C-Term(Methyl)	IP
nTLAnVTFEQk	PF3D7_1364100	6-cysteine protein (P92)	K11(Trimethyl)	IP
EAQMDTTSDDFknVQEFISQMLALLkSYLHVVILIEDHIIKTnLENLLK	PF3D7_1364400	conserved Plasmodium protein, unknown function	K12(Methyl); K26(Methyl)	IP
ENILEILDEEk	PF3D7_1366400	rhoptry protein RHOP148 (RHOP148)	K11(Trimethyl)	HILIC
ETnFYYEnGGQIYDTGIIQNDNMkFQVLNVQK	PF3D7_1367700	alanine–tRNA ligase (AlaRS)	K24(Trimethyl)	IP
YQqSNDTYkQLHELLEK	PF3D7_1372500	stevor, pseudogene	K9(Methyl)	HILIC
SSLPGSIqk	PF3D7_1373400	rifin (RIF)	K9(Dimethyl)	IP
FcLTQNGSAGGGGSGnASGSSGDcGGGNSDSSLCEPWk	PF3D7_1373500	erythrocyte membrane protein 1, PfEMP1 (VAR)	K38(Trimethyl)	HILIC
IHIFLNnENMDELIkNNILELSFGLHFGWAIEGAIGSSYK	PF3D7_1404600.2	adenylyl cyclase alpha (ACalpha)	K15(Trimethyl)	IP
IDHIcYnSTNESEGk	PF3D7_1407600	conserved Plasmodium protein, unknown function	K15(Dimethyl)	HILIC
MNINEkDKLAEQNLETLDVTK	PF3D7_1410600	eukaryotic translation initiation factor 2 gamma subunit, putative	K6(Trimethyl)	IP
MNINEKDkLAEQNLETLDVTK	PF3D7_1410600	eukaryotic translation initiation factor 2 gamma subunit, putative	K8(Trimethyl)	IP
MDMLNPqFEEIGkEFVNHYFQLFNSGR	PF3D7_1412300	nuclear transport factor 2, putative (NTF2)	K13(Trimethyl)	HILIC
cINNLCLnLkNDDIDYYINCk	PF3D7_1412400	conserved Plasmodium protein, unknown function	K10(Trimethyl); K21(Trimethyl)	IP
AIGAGAGAGAGAGSASASGGQLMk	PF3D7_1414100	conserved Plasmodium protein, unknown function	K24(Methyl)	HILIC
SNHGNLISWSEQGVFLLnTSLTVEENkPASHKNYGWETFTDTVINIINRQK	PF3D7_1415000	uracil-DNA glycosylase (UDG)	K27(Methyl)	IP
SNIIITTSNNAk	PF3D7_1416200	metacaspase-like protein (MCA3)	K12(Trimethyl)	HILIC
MLYIFFNNNLLNLFk	PF3D7_1420500	conserved Plasmodium membrane protein, unknown function	C-Term(Methyl)	HILIC
NDDInnFnSkIIEGQVLFDK	PF3D7_1420600	pantothenate kinase, putative (PANK)	K10(Dimethyl)	HILIC
FSGCPAHFHNINYnLCDEEk	PF3D7_1421100	conserved Plasmodium protein, unknown function	C-Term(Methyl)	IP
TLqNqEIIHNNDTNQkNNKVSSINKINTNK	PF3D7_1422700	conserved Plasmodium protein, unknown function	K16(Trimethyl)	IP
NDPnIknILNK	PF3D7_1426100	basic transcription factor 3b, putative	K6(Methyl);	HILIC
MEPTTETkTEEIDLEK	PF3D7_1428300	proliferation-associated protein 2g4, putative	K8(Trimethyl)	HILIC
NkDITKYNNNNNDNNNNDNnnnDNNNNDNKNGCDNIK	PF3D7_1429400	rRNA (adenosine-2’-O-)-methyltransferase, putative	K2(Dimethyl)	IP
ASIqNISSEqk	PF3D7_1430800	conserved Plasmodium protein, unknown function	C-Term(Methyl)	IP
nETDHNLFWTCcSEENVEkPR	PF3D7_1432200	conserved Plasmodium protein, unknown function	K19(Dimethyl)	HILIC
NSScNkqTHMNCVQENYMTQnSSFDILSTEK	PF3D7_1433200	conserved Plasmodium protein, unknown function	K6(Trimethyl)	IP
EDKDDAGqDnqkWEDDIK	PF3D7_1434500	dynein-related AAA-type ATPase, putative	K12(Trimethyl)	IP
nSVSAYnGPEFEDLDDSLQTSLDEWLANLGVDSELCDFIDSCSIDk	PF3D7_1434800	mitochondrial acidic protein MAM33, putative	K46(Trimethyl)	HILIC
SDMTDDnYIDVSEkHVIILGGGKTAVDCISLAIr	PF3D7_1435300	NAD(P)H-dependent glutamate synthase, putative	K14(Dimethyl)	IP
VSTISAcEkSDVnVTTNGISRcHEDK	PF3D7_1435600	conserved Plasmodium protein, unknown function	K9(Methyl)	IP
IPNIGScYFVIIGHMkPIQLYVIINELQSTYnNLDEIIILTSLPIk	PF3D7_1436100	conserved Plasmodium membrane protein, unknown function	K16(Trimethyl); K46(Dimethyl)	HILIC
ATFEGNVDDDDTVYDEnEEnYDCLkk	PF3D7_1438100	secretory complex protein 62 (SEC62)	K25(Dimethyl); K26(Dimethyl)	IP
FTENYNFALLPQDHSk	PF3D7_1438400	metacaspase-like protein (MCA2)	C-Term(Methyl)	HILIC
knVSGnINMPSFVK	PF3D7_1438700	DNA primase small subunit	K1(Dimethyl)	IP
FLVQNKcLEVVQENYVLNYYATPLGHIASMYYIkCETVYFFYTSIQAGk	PF3D7_1439100	DEAD/DEAH box helicase, putative	K34(Trimethyl); K49(Trimethyl)	IP
LISnNDSkDELFSNLNR	PF3D7_1440000	conserved Plasmodium protein, unknown function	K8(Methyl)	IP
MEknKDMENLKNADILK	PF3D7_1440500	allantoicase, putative	K3(Trimethyl)	IP
EMINEYkTNkLnDSNIALMIEEKK	PF3D7_1440600	conserved Plasmodium protein, unknown function	K7(Dimethyl); K10(Trimethyl)	IP
EEVTQIDIINnkEQNISSNSVIk	PF3D7_1442700	conserved Plasmodium protein, unknown function	K12(Dimethyl); K23(Trimethyl)	IP
ISnLTDVNTNIk	PF3D7_1444100	conserved Plasmodium protein, unknown function	K12(Dimethyl)	IP
INYDVMIEDFDEnnk	PF3D7_1444100	conserved Plasmodium protein, unknown function	C-Term(Methyl)	IP
NPNFYICGNGYIAPLDIk	PF3D7_1444200	calmodulin-like protein	K18(Dimethyl)	HILIC
ALQEnGVLLEGALLkPNMVTAGYEcTAK	PF3D7_1444800	fructose-bisphosphate aldolase (FBPA)	K15(Trimethyl);	HILIC
NEAGVPMVnLLHnENIIPGIk	PF3D7_1444800	fructose-bisphosphate aldolase (FBPA)	K21(Methyl)	HILIC
NNNIVAPTPIqIqGWPIALSGk	PF3D7_1445900	ATP-dependent RNA helicase DDX5, putative (DDX5)	K22(Methyl)	HILIC
EKnnKEEnVVk	PF3D7_1446500	conserved Plasmodium protein, unknown function	K11(Dimethyl)	IP
TNIISNHLLDIQNVNSVQkLqSHVPHSYGNHISNGcSENTGLTYSk	PF3D7_1450300	NADPH–cytochrome P450 reductase, putative (CPR)	K19(Methyl); K46(Dimethyl)	IP
WNDInINLNMcDVMPCNk	PF3D7_1450800	conserved Plasmodium protein, unknown function	K18(Trimethyl)	IP
ETVTEESTITcLGk	PF3D7_1451100	elongation factor 2 (eEF2)	K14(Methyl)	HILIC
GYVQPVSLTSSDYFHSCYTSSYESQMFDCnAGFSGnnQEk	PF3D7_1451600	LCCL domain-containing protein (LAP5)	K40(Methyl)	HILIC
VCPkINNDNVLKcEFEESSLYNPk	PF3D7_1452000	rhoptry neck protein 2 (RON2)	K4(Trimethyl); C-Term(Methyl)	IP
EIYDKqQINk	PF3D7_1453800	glucose-6-phosphate dehydrogenase-6-phosphogluconolactonase (GluPho)	K10(Dimethyl)	IP
YQEITDkSnTVDDASK	PF3D7_1457000	signal peptide peptidase (SPP)	K7(Trimethyl)	HILIC
NIAFYnLVIGNNNk	PF3D7_1457400	conserved Plasmodium protein, unknown function	K14(Trimethyl)	HILIC
DHPTVHCFTNGDnFLYDTGkIIqDk	PF3D7_1460400	ubiquitin carboxyl-terminal hydrolase isozyme L3 (UCHL3)	K20(Dimethyl); C-Term(Methyl)	IP
NGICPNIDnTLMLVEDSLk	PF3D7_1461800	conserved Plasmodium protein, unknown function	C-Term(Methyl)	HILIC
NMLPCIENTNTSLNrANQNHFDkNEVr	PF3D7_1461800	conserved Plasmodium protein, unknown function	K23(Trimethyl)	IP
HNLNYINIFTLDGHINENGGk	PF3D7_1461900	valine–tRNA ligase, putative	C-Term(Methyl)	IP
STILLPETYFKPFIkPLQHSSIINHNCLINInEQLFnYFLFLFFNk	PF3D7_1462100	conserved Plasmodium protein, unknown function	K15(Dimethyl); K46(Dimethyl)	IP
KNDTNNQTnDLNSNkDETSGSQVTNR	PF3D7_1462300	conserved Plasmodium protein, unknown function	K15(Trimethyl)	HILIC
VSVFAEkDPSqIPWGK	PF3D7_1462800	glyceraldehyde-3-phosphate dehydrogenase (GAPDH)	K7(Trimethyl);	HILIC
NSDTNSNIMSNTYEnMYEDFSSnYDDSAck	PF3D7_1464500	conserved Plasmodium membrane protein, unknown function	K30(Dimethyl)	HILIC
kPnIAnK	PF3D7_1466200	conserved Plasmodium protein, unknown function	K1(Methyl)	HILIC
IDELInIFk	PF3D7_1467100	DNA-3-methyladenine glycosylase, putative	K9(Dimethyl)	HILIC
ELNENEIkGnHR	PF3D7_1467900	rab GTPase activator, putative	K8(Dimethyl);	HILIC
EIWDQcTIAVYNnTLNAVESkPLLFLHGILNECr	PF3D7_1471100	exported protein 2 (EXP2)	K21(Trimethyl)	IP
ELNNFHFYLIVkINnLnIVPYLNLYMCr	PF3D7_1471900	conserved Plasmodium protein, unknown function	K12(Methyl); C-Term(Methyl)	HILIC
ELVNIQMnDkInEK	PF3D7_1474300	DNA repair metallo-beta-lactamase protein, putative	K10(Trimethyl);	HILIC
CLGTLINLcSDGSTGYqcNNCSk	PF3D7_1475400	cysteine repeat modular protein 4 (CRMP4)	K23(Trimethyl)	HILIC
cGFGLSGVAGSIGLFGAVAInIWkPAALKAAIAKAITEGTADIAAAGVkAGEVTGK	PF3D7_1479400	rifin (RIF)	K24(Trimethyl); K49(Dimethyl)	IP
GcLRcGSILGAAMPELGSVGGSLLYALNTWKPAAIIAAkEAALAEATDLATQAGIDTVVAQLk	PF3D7_1479700	rifin (RIF)	K39(Trimethyl); K63(Trimethyl)	IP

The sequences in bold represent two inner membrane complex proteins (IMC1c and IMC1g) for which a large number of lysine methylation sites were identified.
