# Supplementary material for: Widespread occurrence of lysine methylation in Plasmodium falciparum proteins at asexual blood stages
Source: Sci Rep. 2016 Oct 20;6:35432. doi: 10.1038/srep35432 (PMC5071865; doi:10.1038/srep35432)

Widespread occurrence of lysine methylation of *Plasmodium falciparum* proteins at asexual blood stages

Inderjeet Kaur*[1], Mohammad Zeeshan*[1,2], Ekta Saini[1], Abhinav Kaushik[2], Asif Mohmmed[3], Dinesh Gupta[§2] and Pawan Malhotra[§1]

[1]Malaria Biology Group, International Centre for Genetic Engineering and Biotechnology, ICGEB, Aruna Asaf Ali Marg, New Delhi – 110067, INDIA

[2]Translational Bioinformatics Group, International Centre for Genetic Engineering and Biotechnology, Aruna Asaf Ali Marg, New Delhi-110067, India

[3]Parasite Cell Biology Group, International Centre for Genetic Engineering and Biotechnology, Aruna Asaf Ali Marg, New Delhi-110067, India

Tel. +91-11-26741358; Fax: +91-11-26742316;

* authors have contributed equally.

[§]To whom correspondence should be addressed. E-mail: pawanm@icgeb.res.in; dinesh@icgeb.res.in

Supplementary Figure 1: The representative spectra of *Plasmodium* lysine-methylated peptides identified by IP-LC-MS/MS.

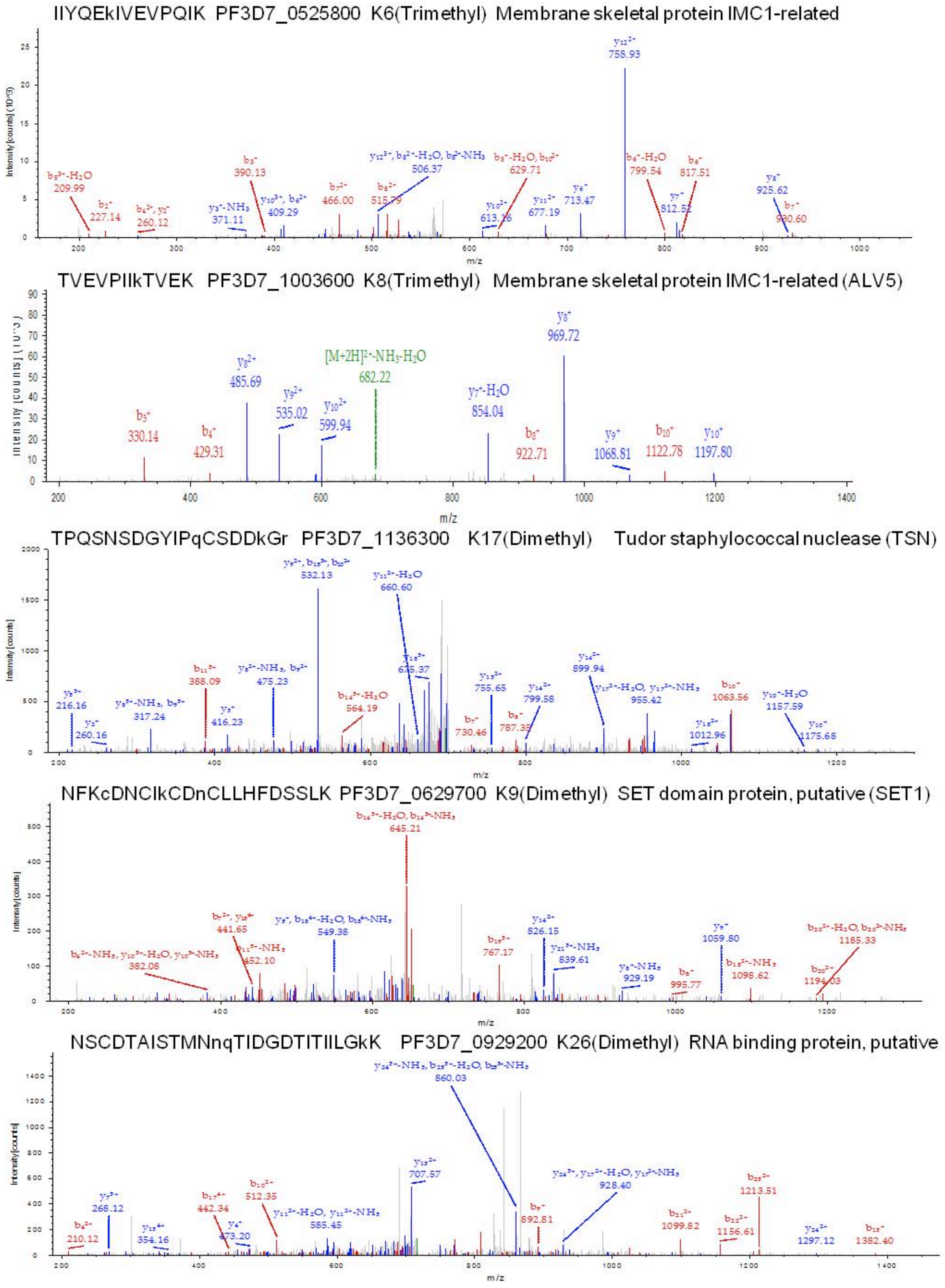

Supplementary Figure 2: The representative spectra of *Plasmodium* lysine-methylated peptides identified by HILIC and LC-MS/MS.

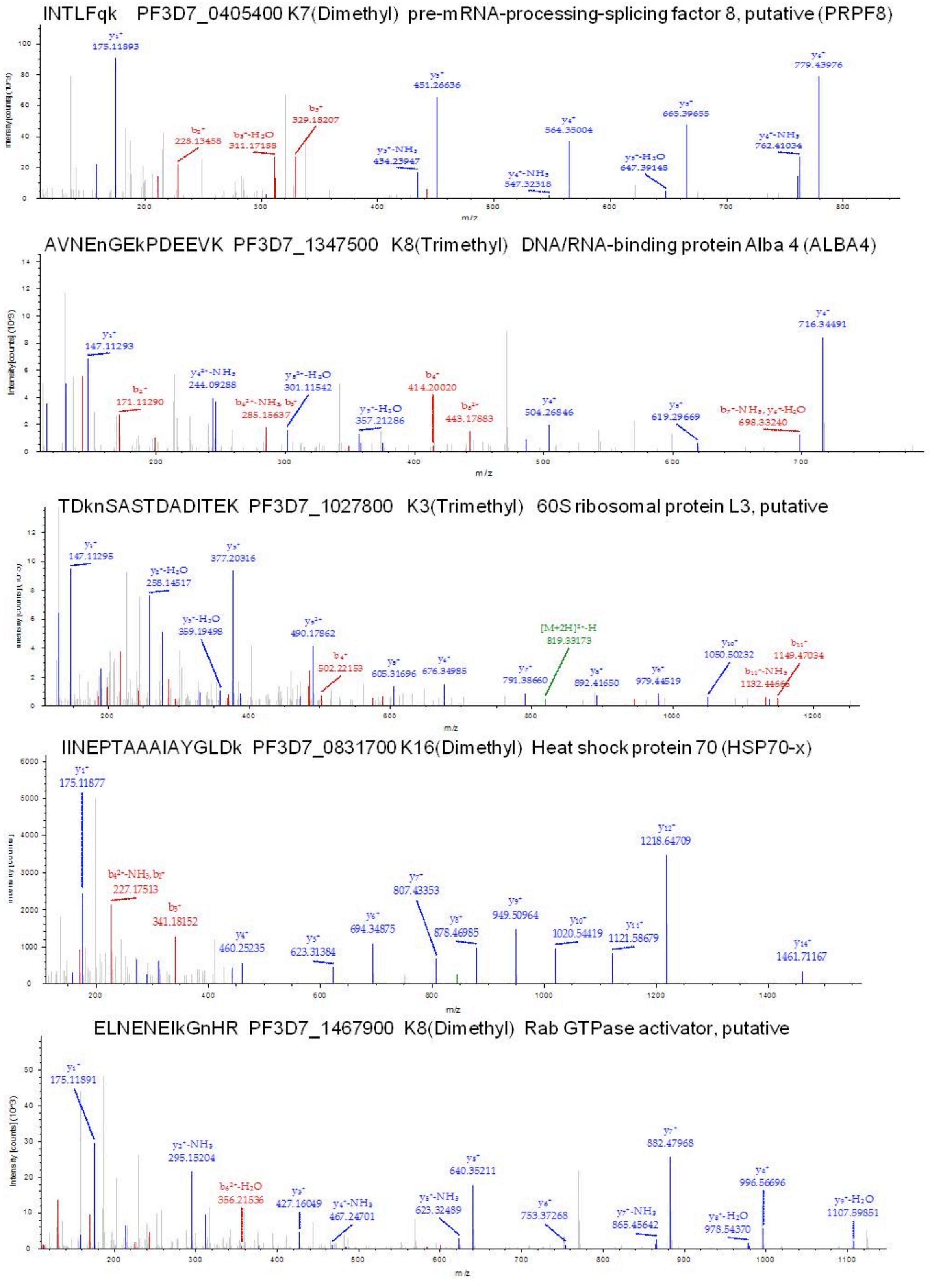

Supplementary Figure 3: Interactome analysis of PfSET domain proteins using STRING database.

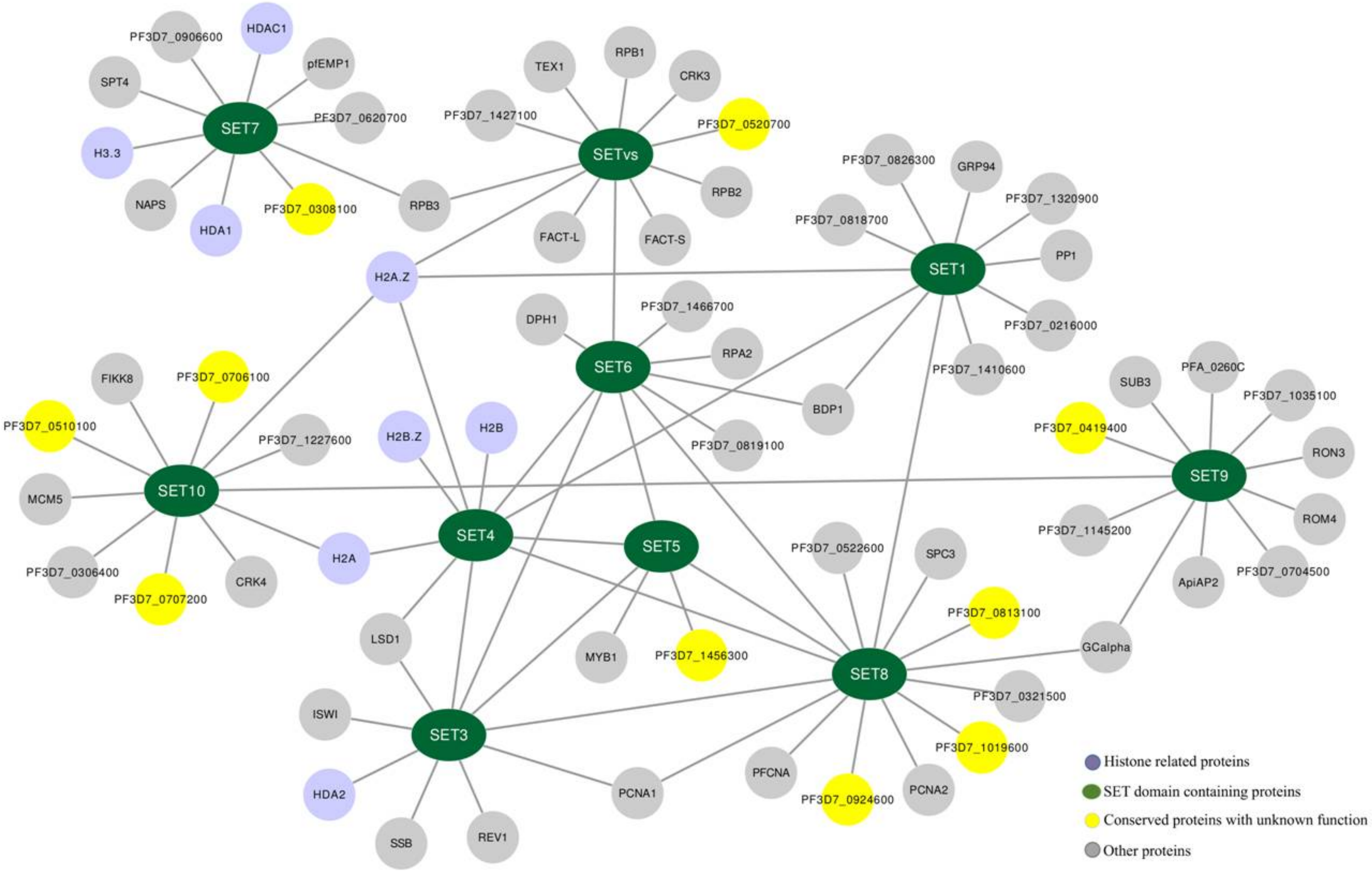

Supplementary Figure 4: The "Two Sample Logo" representation of the residues surrounding methylated and non methylated lysines.

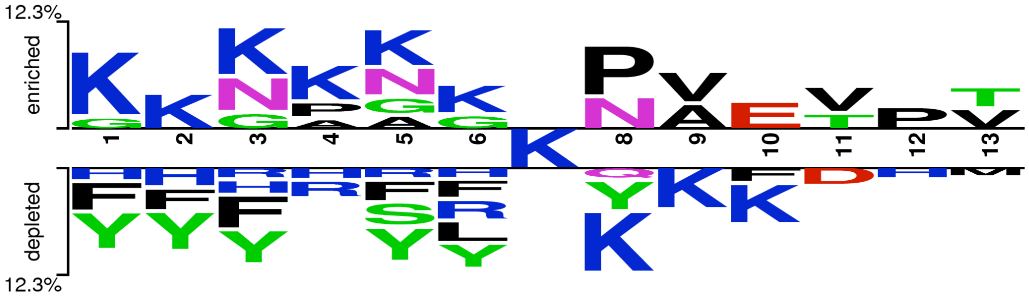

Supplement: Supplementary Information [file srep35432-s3.pdf]
